# Validating a 14-Drug Microtiter Plate Containing Bedaquiline and Delamanid for Large-Scale Research Susceptibility Testing of Mycobacterium tuberculosis

**DOI:** 10.1128/AAC.00344-18

**Published:** 2018-08-27

**Authors:** Paola M. V. Rancoita, Federica Cugnata, Ana Luíza Gibertoni Cruz, Emanuele Borroni, Sarah J. Hoosdally, Timothy M. Walker, Clara Grazian, Timothy J. Davies, Timothy E. A. Peto, Derrick W. Crook, Philip W. Fowler, Daniela M. Cirillo, for the CRyPTIC Consortiumfor the CRyPTIC Consortiumfor the CRyPTIC Consortium, Derrick W. Crook

**Affiliations:** University of Oxford; University of Oxford; University of Oxford; University of Oxford; University of Oxford; University of Oxford; University of Oxford; University of Oxford; University of Oxford; University of Oxford; European Bioinformatics Institute; European Bioinformatics Institute; Public Health England, Birmingham, United Kingdom; Public Health England, Birmingham, United Kingdom; Public Health England, Birmingham, United Kingdom; Public Health England, Birmingham, United Kingdom; Emerging Bacterial Pathogens Unit, IRCCS San Raffaele Scientific Institute, Milan, Italy; Emerging Bacterial Pathogens Unit, IRCCS San Raffaele Scientific Institute, Milan, Italy; Emerging Bacterial Pathogens Unit, IRCCS San Raffaele Scientific Institute, Milan, Italy; Emerging Bacterial Pathogens Unit, IRCCS San Raffaele Scientific Institute, Milan, Italy; Emerging Bacterial Pathogens Unit, IRCCS San Raffaele Scientific Institute, Milan, Italy; Emerging Bacterial Pathogens Unit, IRCCS San Raffaele Scientific Institute, Milan, Italy; National Tuberculosis Control Program Pakistan, Islamabad, Pakistan; The Foundation for Medical Research, Mumbai, India; The Foundation for Medical Research, Mumbai, India; The Foundation for Medical Research, Mumbai, India; The Foundation for Medical Research, Mumbai, India; The Foundation for Medical Research, Mumbai, India; P. D. Hinduja National Hospital and Medical Research Centre, Mumbai, India; P. D. Hinduja National Hospital and Medical Research Centre, Mumbai, India; P. D. Hinduja National Hospital and Medical Research Centre, Mumbai, India; P. D. Hinduja National Hospital and Medical Research Centre, Mumbai, India; Research Center Borstel; Research Center Borstel; Research Center Borstel; Institute of Microbiology & Laboratory Medicine, IML Red, Gauting, Germany; Institute of Microbiology & Laboratory Medicine, IML Red, Gauting, Germany; Institute of Microbiology & Laboratory Medicine, IML Red, Gauting, Germany; National Institute for Communicable Diseases, Johannesburg, South Africa; National Institute for Communicable Diseases, Johannesburg, South Africa; National Institute for Communicable Diseases, Johannesburg, South Africa; National Institute for Communicable Diseases, Johannesburg, South Africa; Oxford University Clinical Research Unit, Ho Chi Minh City, Vietnam; Oxford University Clinical Research Unit, Ho Chi Minh City, Vietnam; Oxford University Clinical Research Unit, Ho Chi Minh City, Vietnam; Oxford University Clinical Research Unit, Ho Chi Minh City, Vietnam; London School of Hygiene and Tropical Medicine and Universidad Peruana Cayetano Heredía, Lima, Peru; London School of Hygiene and Tropical Medicine and Universidad Peruana Cayetano Heredía, Lima, Peru; London School of Hygiene and Tropical Medicine and Universidad Peruana Cayetano Heredía, Lima, Peru; China CDC, Beijing, China; China CDC, Beijing, China; China CDC, Beijing, China; China CDC, Beijing, China; China CDC, Beijing, China; The Critical Path Institute, Tucson, AZ; Scottish Mycobacteria Reference Laboratory; Scottish Mycobacteria Reference Laboratory; a University Centre of Statistics in the Biomedical Sciences, Vita-Salute San Raffaele University, Milan, Italy; b Emerging Bacterial Pathogens Unit, Division of Immunology, Transplantation and Infectious Diseases, IRCCS San Raffaele Scientific Institute, Milan, Italy; c Nuffield Department of Medicine, John Radcliffe Hospital, University of Oxford, Headley Way, Oxford, United Kingdom

**Keywords:** Mycobacterium tuberculosis, antibiotic resistance, antimicrobial agents, diagnostics

## Abstract

The UKMYC5 plate is a 96-well microtiter plate designed by the CRyPTIC Consortium (Comprehensive Resistance Prediction for Tuberculosis: an International Consortium) to enable the measurement of MICs of 14 different antituberculosis (anti-TB) compounds for >30,000 clinical Mycobacterium tuberculosis isolates. Unlike the MYCOTB plate, on which the UKMYC5 plate is based, the UKMYC5 plate includes two new (bedaquiline and delamanid) and two repurposed (clofazimine and linezolid) compounds. UKMYC5 plates were tested by seven laboratories on four continents by use of a panel of 19 external quality assessment (EQA) strains, including H37Rv. To assess the optimal combination of reading method and incubation time, MICs were measured from each plate by two readers, using three methods (mirrored box, microscope, and Vizion digital viewing system), after 7, 10, 14, and 21 days of incubation. In addition, all EQA strains were subjected to whole-genome sequencing and phenotypically characterized by the 7H10/7H11 agar proportion method (APM) and by use of MGIT960 mycobacterial growth indicator tubes. We concluded that the UKMYC5 plate is optimally read using the Vizion system after 14 days of incubation, achieving an interreader agreement of 97.9% and intra- and interlaboratory reproducibility rates of 95.6% and 93.1%, respectively. The mirrored box had a similar reproducibility. Strains classified as resistant by APM, MGIT960, or the presence of mutations known to confer resistance consistently showed elevated MICs compared to those for strains classified as susceptible. Finally, the UKMYC5 plate records intermediate MICs for one strain for which the APM measured MICs close to the applied critical concentration, providing early evidence that the UKMYC5 plate can quantitatively measure the magnitude of resistance to anti-TB compounds that is due to specific genetic variation.

## Abstract (precis)

The UKMYC5 plate is a 96-well microtiter plate designed by the CRyPTIC Consortium (Comprehensive Resistance Prediction for Tuberculosis: an International Consortium) to enable the measurement of MICs of 14 different antituberculosis (anti-TB) compounds for >30,000 clinical Mycobacterium tuberculosis isolates. Unlike the MYCOTB plate, on which the UKMYC5 plate is based, the UKMYC5 plate includes two new (bedaquiline and delamanid) and two repurposed (clofazimine and linezolid) compounds.

## INTRODUCTION

The proportion of tuberculosis (TB) cases that are multidrug resistant (MDR) is increasing worldwide. Although set against a background of a falling global incidence of TB, the net effect is that the number of MDR-TB cases continues to grow (1). Improving the treatment success rate for MDR-TB requires each patient to receive an individual antimicrobial regimen tailored to maximize efficacy while minimizing toxicity; this necessitates being able to measure MICs to direct both the choice of drug and the dose. Universal access to prompt and comprehensive drug susceptibility testing (DST) is therefore a key component of the WHO's “End TB” strategy (2, 3). Although molecular approaches have the potential to deliver universal DST methods, they require further development work, and any resulting solutions are likely to be expensive.

Liquid- and solid-medium assays that measure MICs for TB exist (4–9) but are time-consuming and often costly, and so far they are not yet endorsed by any international regulatory authorities or other international health organizations, such as the WHO. Microtiter plates offer a way of testing, in parallel, the effectiveness of a large number of drugs at a range of concentrations on small aliquots taken from a single clinical isolate. Broth microdilution methods, including several using colorimetric indicators, have previously been developed that assess the MICs of a panel of compounds by using a single microtiter plate (5, 10, 11). A dry-format, 96-well microtiter plate assay (the Sensititre MYCOTB plate; Thermo Fisher Scientific Inc., USA) containing 12 drugs has been commercially available since 2010, and early validation studies have returned promising results (12–17). At present, however, neither clinical breakpoints nor epidemiological cutoffs have been defined for any microtiter plate, nor have any plate-based assays included both new and repurposed drugs that will be key to the successful treatment of individual MDR-TB cases in the future.

We designed the UKMYC5 plate, which is a variant of the MYCOTB plate, to enable the CRyPTIC Consortium (Comprehensive Resistance Prediction for Tuberculosis: an International Consortium) to measure the MICs of 14 different anti-TB compounds (see Fig. S1 and Table S1 in the supplemental material) for a large number (>30,000) of clinical TB isolates that are being collected globally by participating laboratories between 2017 and 2020. Each isolate will also have its genome sequenced, and the ultimate goals of the CRyPTIC project are (i) to uncover all variations in genes known to be involved in resistance and classify the magnitudes of their effects on specific anti-TB compounds and (ii) to identify new genes that are associated with resistance. The MICs therefore need to be quantitatively reproducible and accurate.

In this paper, we establish the reproducibility and accuracy of the dry-format, 96-well UKMYC5 microtiter plate for use in large-scale measurement of MICs by the CRyPTIC tuberculosis research project. We therefore assess both the reproducibility of MIC measurements obtained using this microtiter plate and their accuracy by comparing the method to a range of established DST methods. Since the UKMYC5 plate contains 14 drugs, including, in contrast to the MYCOTB plate, two new compounds (delamanid and bedaquiline) and two repurposed drugs (clofazimine and linezolid), in the future it may potentially form the basis of a new DST protocol for tailoring regimens to treat individual cases of MDR-TB.

## RESULTS

### Plate design and evaluation process.

The UKMYC5 plate was designed by the CRyPTIC Consortium. The main differences compared to the commercially available MYCOTB 96-well microtiter plate are that bedaquiline, delamanid, clofazimine, and levofloxacin are included and ofloxacin, streptomycin, and cycloserine were removed. The wells for each drug are arranged in doubling dilution series. Since the plate comprises 12 columns of 8 wells and 14 drugs are included, most drugs have 6 (64× concentration range) or 7 wells, with a few having 5 or 8 wells (see Fig. S1 and Table S1 in the supplemental material).

To validate the UKMYC5 plate for use in large-scale research susceptibility testing, it is necessary to demonstrate that the results obtained with the plate are both reproducible and accurate. Unfortunately, no standards for mycobacterial DST exist yet. We therefore borrow, where appropriate, the definitions established for aerobic bacteria. The conclusions we draw about reproducibility and accuracy should therefore be treated as tentative, as they will need revision once a standard for mycobacterial DST is established.

The concentration range for each drug on the UKMYC5 plate is defined by the plate design and therefore fixed. MICs not included in the range are termed “off-scale.” Since MICs are conventionally defined as being in agreement if they lie within a doubling dilution, reproducibility and accuracy are defined exactly only for on-scale growth; including off-scale growth may alter the levels of reproducibility and accuracy.

### Study design.

**FIG 1:**
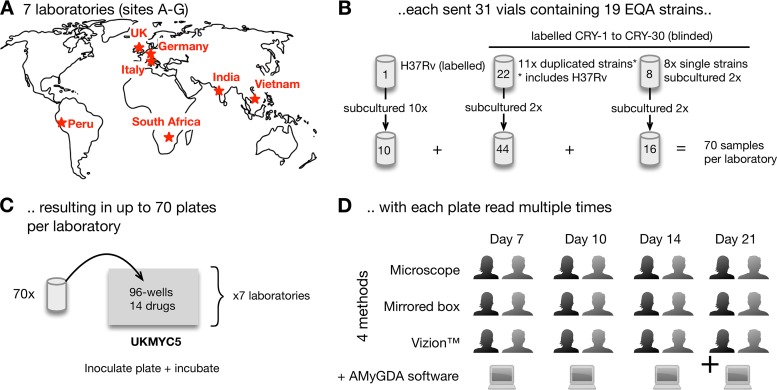
Study design for validating the UKMYC5 plate. (A and B) Seven laboratories (A) were each sent 31 vials (B) containing 19 different genotypically characterized strains. H37Rv ATCC 27294 was subcultured and tested 10 times, while the other strains were subcultured in duplicate. (C) Each strain was inoculated onto a UKMYC5 96-well plate, starting from independent bacterial suspensions. Due to the large number of strains, the culturing and inoculation were performed over a period of weeks in each laboratory. (D) The MICs of each drug were read independently at 7, 10, 14, and 21 days postinoculation by two laboratory scientists, using three methods, as long as the positive-control wells showed acceptable and visible growth. Each plate was also photographed and the image analyzed using AMyGDA software (see Fig. S2 in the supplemental material).

In brief, each of the seven participating laboratories (Fig. 1A) received 19 external quality assessment (EQA) strains, which were selected to ensure that there was at least one strain that was resistant to each of the 14 drugs on the UKMYC5 plate (except for linezolid) (Table S16). Samples from each vial were subcultured either 2 or 10 times (Fig. 1B) and then inoculated onto a UKMYC5 plate (Fig. 1C). Each plate was incubated for 21 days, and two laboratory scientists independently determined the MICs of all 14 anti-TB drugs at four different time points (days 7, 10, 14, and 21) by using a Vizion imaging system, a mirrored box, and an inverted-light microscope (Fig. 1D). In addition, a photograph was taken at each time point by use of the Vizion instrument, and this was retrospectively analyzed using some bespoke plate-reading software, AMyGDA (Fig. S2) (18). For more information on the above, please see Materials and Methods.

### Proportion of readable plates.

**FIG 2:**
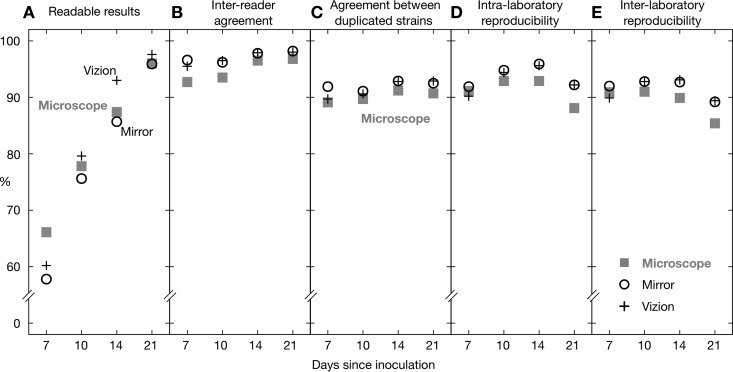
For each reading method, the percentage of readable results (A), interreader agreement (B), agreement between two plates inoculated with different subcultures of the same strain in a single laboratory (C), and intralaboratory (D) and interlaboratory (E) reproducibilities were determined (see Table S2 in the supplemental material). These data include both on- and off-scale MICs. Data from site F were excluded from this analysis.

The proportion of readable results increased with the elapsed time since inoculation. As noted elsewhere (see Materials and Methods), the results for site F were anomalous, and the data from this laboratory were excluded from the study. For the remaining six sites, the proportion of readable results was 57.8 to 66.1% (depending on the reading method) after 7 days of incubation, which then increased to 85.7 to 93.0% after 14 days and reached ≥95.9% after 21 days (Fig. 2A; Table S2). Plates were considered not readable if insufficient growth of Mycobacterium tuberculosis in both positive-control wells was reported. As expected, the proportion of measured MICs that were off-scale, in general, fell as the incubation time increased (Table S4); however, even after 21 days, there remained large differences between drugs, with linezolid having the fewest off-scale readings (0.4 to 0.8%, depending on the reading method) and rifabutin having the most (81.4 to 83.2%, depending on the reading method). The exact pattern for each drug depends on the plate design and how many EQA strains are resistant to its action. A logistic mixed-effects model demonstrated that significantly fewer plates were readable at days 7 and 10 than at day 14 and that significantly more plates were readable at day 21 than at day 14 (*P* < 0.001 for all comparisons) (Table S5). The proportion of results readable by use of an inverted-light microscope or mirrored box was significantly lower than that for the Vizion instrument (*P* < 0.001 in both instances) (Fig. 2A; Table S5).

### Agreement between readers and duplicated strains.

As stated in Materials and Methods, we define two measurements as agreeing if they are within a doubling dilution of one another. The interreader agreement was at least 92.7%, regardless of reading day or method (Fig. 2B; Table S2), and it increased with incubation time (Fig. 2B). For Vizion and mirrored-box imaging, this was ≥95.5% across all reading days, while for the inverted-light microscope it increased from 92.7% at day 7 to 96.8% at day 21 (Fig. 2B). The corresponding statistical model showed that there was significantly less agreement between readers when they used the inverted-light microscope versus the Vizion instrument or when they read the plates at day 7 versus day 14 (*P* < 0.001 for both comparisons) (Table S5). The levels of interreader agreement were similar if we considered only the strain-drug combinations for which the modal MIC was on-scale (Table S3).

As expected, the agreement between MICs measured from two UKMYC5 plates in the same laboratory, each having been inoculated from different cultures of the same strain taken from the same vial (i.e., duplicated strains), was lower, with ≥89.7% of MICs in agreement, regardless of reading day and method (Fig. 2C; Table S2). The maximum values were observed for the Vizion instrument after 14 and 21 days of incubation (92.8 and 92.9%, respectively) and the mirrored box after 14 days of incubation (92.9%). The corresponding logistic mixed-effects model showed that the agreement of the results between duplicated strains was significantly lower at day 7 than at day 14 (*P* < 0.001) and was also lower when the results were obtained with a microscope than when they were obtained with a Vizion instrument (*P* = 0.010) (Table S5).

### Reproducibility.

The intralaboratory reproducibility (the proportion of MICs within a doubling dilution of the mode, including both on- and off-scale readings) was ≥88.1% (Fig. 2D; Table S2), regardless of reading day and method. The maximum values were observed after 14 days of incubation, when the mirrored-box and Vizion intralaboratory reproducibilities were 95.9% and 95.6%, respectively. If we remove, for each drug, the strains for which the modal MIC was off-scale, then the intralaboratory reproducibility increases slightly for all reading days and methods, with maximum values of 96.5% and 96.9% for the mirrored-box and Vizion systems, respectively, at day 14 (Table S3).

The interlaboratory reproducibility was slightly lower, as one would expect, and was ≥85.4% regardless of the reading-day and method combination, peaking again after 14 days, at 93.1% and 92.7% with the Vizion system and the mirrored box, respectively (Fig. 2E; Table S2). Again, for each drug, removing strains for which the modal MIC was off-scale led to a slight increase of the interlaboratory reproducibility at days 14 and 21 for all methods, with the highest values achieved at day 14 by use of the Vizion and mirrored-box systems (94.9% and 94.7%, respectively) (Table S3). The logistic mixed-effects models (considering both on- and off-scale measurements) confirmed that intra- and interlaboratory reproducibilities with the inverted-light microscope were significantly lower, overall, than those with the Vizion system (*P* < 0.001 for all) (Table S6). Moreover, both types of reproducibility were lower at days 7 and 21 than at day 14 (*P* < 0.001 for all) (Table S6).

### Selection of reading method.

Reproducibility and agreement were maximized using either the Vizion system or the mirrored box. The corresponding logistic mixed-effects models could not distinguish between the performances of these two methods. However, the percentages of readable results were higher for the Vizion system than for the mirrored box, and this was confirmed by the corresponding logistic mixed-effects model. Thus, for all subsequent analyses, we selected the Sensititre Vizion digital MIC viewing system. Moreover, this method has the advantage that it records an image of the growth on the UKMYC5 plate, which not only provides a useful audit trail but also permits the use of automated plate-reading software.

### Individual drug performances and selection of reading day.

**FIG 3:**
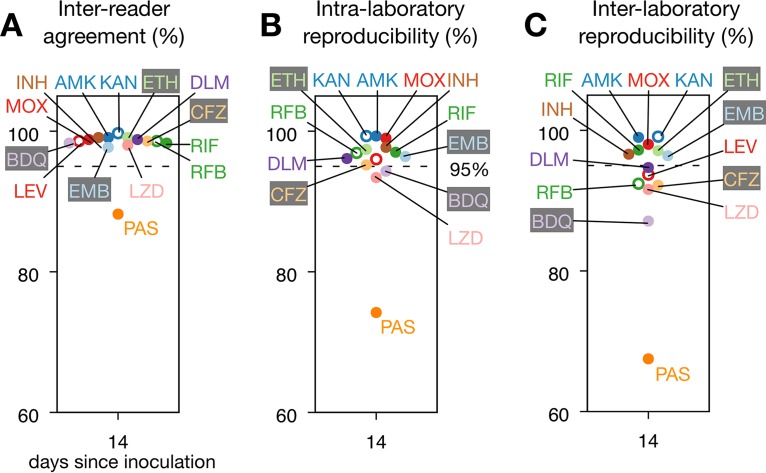
Most drugs performed well on the UKMYC5 plate; however, results for *para*-aminosalicylic acid (PAS) were consistently anomalous. (A) Interreader agreement for the 14 different anti-TB drugs for MICs measured after 14 days of incubation by use of the Vizion system. (B and C) Intralaboratory (B) and interlaboratory (C) reproducibilities. In each graph, a dashed line is drawn at 95%. Results for all reading days can be found in Fig. S3 and Table S7 in the supplemental material. RIF, rifampin; RFB, rifabutin; INH, isoniazid; EMB, ethambutol; LEV, levofloxacin; MOX, moxifloxacin; AMK, amikacin; KAN, kanamycin; ETH, ethionamide; CFZ, clofazimine; PAS, *para*-aminosalicylic acid; LZD, linezolid; DLM, delamanid; BDQ, bedaquiline.

To determine if the individual drugs could be read after 14 days of incubation or whether there was large variation between compounds, we analyzed the individual MICs measured using the Vizion system for each drug and all reading days (Fig. 3; Fig. S3; Table S7). *Para*-aminosalicylic acid (PAS) showed the lowest interreader agreement at each reading day and the lowest intra- and interlaboratory reproducibilities across all reading days, except for day 7 (Fig. S3). This is consistent with a previous study in which PAS had the lowest reproducibility among the 12 drugs on the MYCOTB plate (13). After 14 days of incubation, all 13 remaining anti-TB compounds had values for interreader agreement and intra- and interlaboratory reproducibilities of ≥97.7%, ≥93.4%, and ≥87.1%, respectively. Mirroring the whole-plate trends, the interreader agreement after 21 days tended to be slightly higher (with the exception of that for clofazimine, delamanid, ethionamide, and kanamycin) (Fig. S3), while both measures of reproducibility tended to fall slightly between days 14 and 21, particularly for bedaquiline, delamanid, and, to a lesser extent, ethambutol (Fig. S3). Considering for each drug only the strains with an on-scale MIC mode, most of the above trends are maintained (Table S8), with the greatest difference observed for rifabutin and delamanid, both of which had relatively few on-scale readings, since in both cases only 1 of the 19 strains gave an MIC mode that was on-scale. The results of the corresponding logistic mixed-effects models considering all the measurements (Table S9) showed that, overall, intra- and interlaboratory reproducibilities were significantly lower at days 7 and 21 than at day 14 (*P* = 0.001 and *P* = 0.004, respectively, for day 7 and *P* = 0.001 and *P* < 0.001, respectively, for day 21). The intra- and interlaboratory reproducibilities were indistinguishable between days 10 and 14 (*P* = 0.465 and *P* = 0.784, respectively), and the interreader agreement was lower at day 7 than at day 14 (*P* = 0.030).

Using the same model, reproducibility using the Vizion system was also assessed on an individual drug basis, using rifampin as a comparator selected on the basis of a known and characterized mutation in the *rpoB* gene (Ser450Leu) that leads to high MICs clearly distinguishable from those for wild-type genotypes (Table S9). Although there were statistically significant differences between many of the drugs and rifampin, the greatest difference was seen for *para*-aminosalicylic acid, for which the interreader agreement and the intra- and interlaboratory reproducibilities were both significantly lower than those for rifampin (*P* < 0.001). In general, although the intra- and interlaboratory reproducibilities of the drugs were similar after 10 and 14 days (Table S9), since more results were readable at day 14 than at day 10 (Fig. 2), we elected to read the plates after 14 days of incubation.

### Automated plate reading using AMyGDA software.

Automated plate-reading software offers the ability to add a layer of quality control to plate reading, potentially increasing both reproducibility and accuracy. MICs measured from photographs of the UKMYC5 plates after 14 days of incubation by use of AMyGDA software (Fig. S2) (18) were then compared to MICs measured by the laboratory scientist by use of the Vizion system. As before, we consider the MICs to be in agreement if they are within a doubling dilution of one another. The agreement between the two methods started at 87.9%, at day 7, and increased with incubation time, reaching 93.8% after 21 days (Fig. S4; Table S10). At day 14, the agreement between readings by AMyGDA and the Vizion system was ≥90% for all drugs, except for moxifloxacin (89.3%) and *para*-aminosalicylic acid (73.8%). Since neither technique is an established reference method, we interpret the agreement here to measure the consistency of human- and software-based reading methods. Using a logistic mixed-effects model, we found that the agreement at days 7, 10, and 21 was not significantly different from the agreement at day 14 (*P* = 0.143, *P* = 0.479, and *P* = 0.525, respectively) (Table S11). We later discuss the potential for software, such as AMyGDA, to further improve how microtiter plates are read.

### MIC distributions for H37Rv and agreement with endorsed methods.

Having determined the optimal time and method to read the UKMYC5 plate and examined the reproducibility of this approach, we next assessed the concordance between the UKMYC5 plate and several endorsed methods. We first compared our MIC distributions for the H37Rv reference strain ATCC 27294, as measured by the Vizion system after 14 days of incubation, with MICs measured using (i) a frozen Thermo Fisher custom-made 96-well microtiter plate (15, 16), (ii) the agar proportion method (APM), and (iii) the resazurin microtiter assay (REMA). The modes of the MICs obtained from the frozen microtiter plate were taken from published studies (15, 16, 19). For both APM and REMA, the modes of in-house measurements were taken for each drug (with the exception of ethionamide and PAS, which were not evaluated).

**FIG 4:**
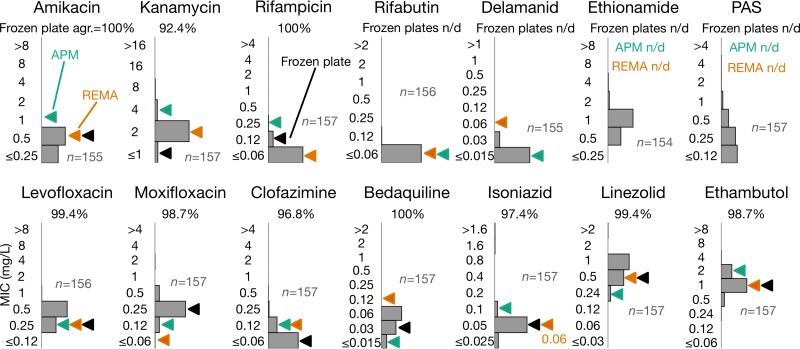
MIC distributions for H37Rv for the 14 drugs on the UKMYC5 plate, as measured at day 14 by use of the Vizion system, are plotted as bar charts in gray. Each drug is annotated with the number of total measurements (*n*). These distributions are compared to MICs measured using three different methods. First, the published mode of the MIC for each drug (where available), measured using a frozen microtiter plate, is indicated by a black filled triangle (15, 16). The agreement with the MICs measured by use of the UKMYC5 plate is given for each drug. Next, the modal MICs measured by the agar proportion method (APM; teal triangles) and the resazurin microtiter assay (REMA; orange triangles) were plotted. n/d, not done.

For the majority of drugs, there was a good level of agreement between the distribution of MICs measured using the UKMYC5 plate and the three comparator measurements (Fig. 4). The agreement between the UKMYC5 modal MICs and the modal MICs taken from the previously published frozen-form microtiter plate study was ≥96.8% for evaluated drugs (i.e., bedaquiline, isoniazid, clofazimine, rifampin, levofloxacin, moxifloxacin, amikacin, linezolid, and ethambutol), with the exception of kanamycin, whose agreement was 92.4% (Fig. 4; Table S12). Moreover, five of the drugs had an agreement of ≥99.4%. We caution, however, that since the modal MICs for four of the drugs (rifampin, rifabutin, delamanid, and clofazimine) were off-scale, the “true” MICs may be more than one doubling dilution from the modes obtained by the other methods, which would reduce the level of agreement. We note that after 21 days of incubation, the agreement between these methods had decreased to 65.2% and 83.0% for kanamycin and ethambutol, respectively (Table S12), providing further reason for preferring 14 days of incubation over 21 days.

### Comparing MIC distributions for all 19 EQA strains with those obtained by APM and MGIT.

**FIG 5:**
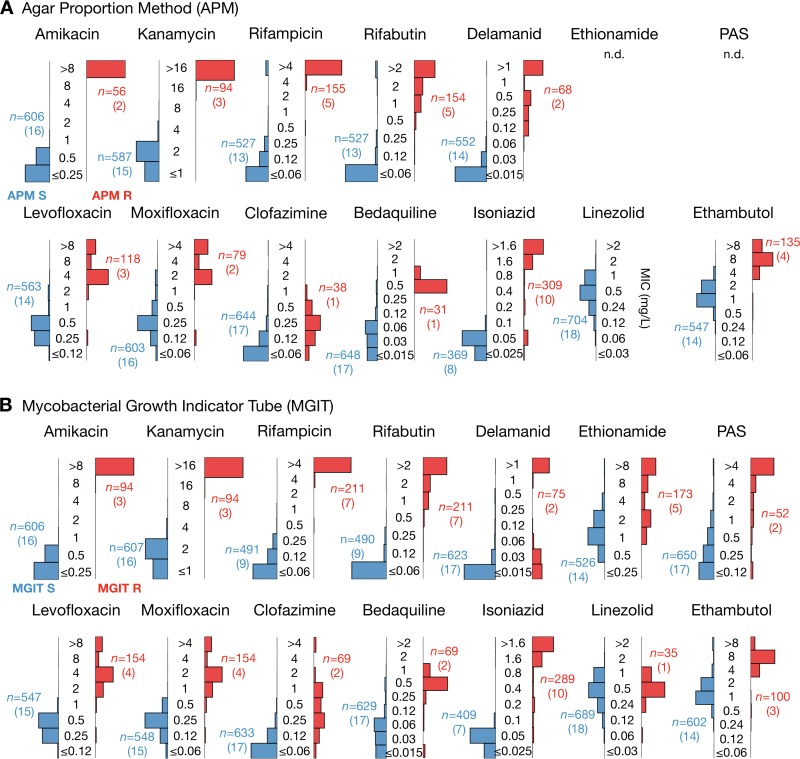
Strains identified as resistant by the agar proportion method (APM) (A) or the mycobacterial growth indicator tube method (MGIT) (B) consistently recorded elevated MICs on the UKMYC5 plate, according to the Vizion instrument, after 14 days of incubation. The 19 EQA strains were categorized as either susceptible or resistant by using either APM or MGIT results. The distributions of MICs (milligrams per liter) measured by all laboratories in this study (excluding those from site F) for the susceptible and resistant strains were then plotted in blue and red, respectively. The critical concentrations used for APM are reported in Table S13 in the supplemental material. The number of UKMYC5 measurements (*n*) for each bar chart is given, with the number of EQA strains provided in parentheses.

Since no critical concentrations (CCs) exist for any microtiter plate, all 19 EQA strains (including H37Rv) were then categorized as resistant or susceptible by using the same CCs as those for the comparator methods (as done previously [13, 14]), which were (i) the 7H10/7H11 agar proportion method (APM) and (ii) the mycobacterial growth indicator tube method (MGIT) (Table S22). Where possible, critical concentrations for APM were taken from recent guidance (20). The MIC distributions measured using the UKMYC5 plate were then plotted separately for the so-defined phenotypically resistant and susceptible strains for each drug (Fig. 5). For most of the drugs, resistant and susceptible strains had clearly different MIC distributions on the UKMYC5 plate (except for linezolid, which showed no resistant strains; clofazimine, *para*-aminosalicylic acid, and ethionamide were not tested). We did not observe the lack of accuracy for ethambutol observed in previous studies using the MYCOTB plate (17, 21). Since APM and MGIT generally classified the same strains as resistant, similar distributions were observed when MGIT was used to classify the strains (Fig. 5B). The small differences between APM and MGIT arose from strains that were classified as resistant by one method and as susceptible by the other. For example, MGIT classified an additional strain, which according to APM had an MIC one doubling dilution below the critical concentration, as resistant to rifampin.

To enable quantitative comparisons to be made between the UKMYC plate and both APM and MGIT, the mode of each UKMYC5 MIC distribution was calculated by drug for each strain and then compared with the categorical results (susceptible or resistant) obtained by MGIT and APM for that strain (13, 22, 23). The categorical and conditional agreements between UKMYC5 MIC modes and those obtained by MGIT and APM were thereby computed (Table S13). Since no critical concentrations exist for the UKMYC5 plate, we assumed shared breakpoints with each comparator method (APM or MGIT) to infer whether each strain was “susceptible” or “resistant” (13, 14). Discrepancies are listed in Table S14; in the majority of cases, the UKMYC5 mode was within one or two doubling dilutions of the assumed critical concentration. This may be due to the fact that the CCs applied are not defined for the UKMYC5 plate. With formally defined CCs, these strains may have been classified correctly.

### Comparison of genotype and UKMYC5 results.

**FIG 6:**
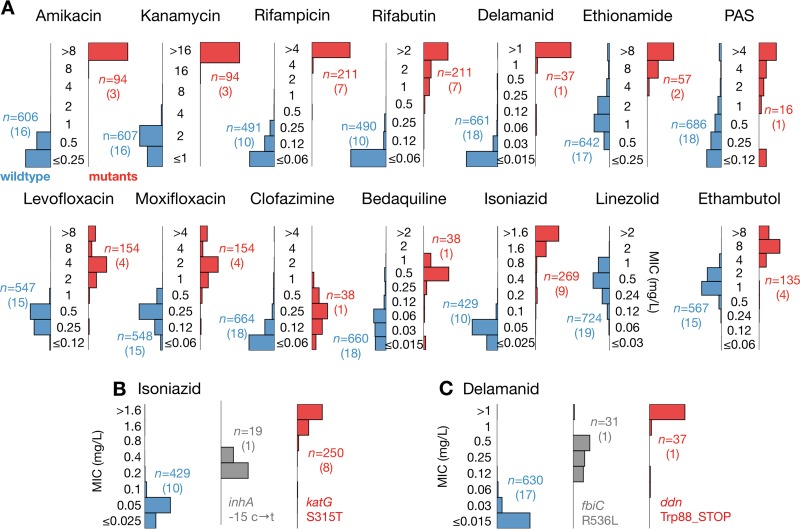
The UKMYC5 plate not only registers higher MICs for strains containing known resistance-conferring genetic variation but also reports intermediate MICs for two strains containing mutations that confer intermediate resistance to delamanid and isoniazid. (A) UKMYC5 MIC distribution for each drug, split by whether the strain contains (red) or does not contain (blue) mutations that confer resistance. Drugs that share the same resistance genes are paired. The list of genetic variants used to make this classification can be found in Table S15 in the supplemental material. The number of UKMYC5 measurements (*n*) for each bar chart is given, with the number of EQA strains provided in parentheses. (B) Intermediate isoniazid MICs were consistently recorded on the UKMYC5 plate for one strain containing a mutation in the promoter of *inhA*. (C) Intermediate delamanid MICs were consistently recorded on the UKMYC5 plate for one strain containing the Arg563Leu mutation encoded within the *fbiC* gene.

Since the genomes of all 19 EQA strains, one of which is H37Rv, are known, one can carry out a comparison between strains harboring variations in certain genes known to confer resistance to a specific compound and those with either no variation or variations with an unknown effect or known to have no effect. For all drugs, in general, strains with genetic mutations accepted to confer phenotypic resistance to specific drugs were associated with elevated UKMYC5 MICs compared to those for strains with no such mutations (Fig. 6A; Table S15) (24).

This was most evident for the second-line injectable drugs (SLID; amikacin and kanamycin) and the rifamycins (rifampin and rifabutin). For the SLID, 3 of the 19 EQA strains contained the well-known A1401G mutation in the *rrs* gene (these strains were also identified as resistant by APM and MGIT). For the rifamycins, 7 of the EQA strains contained a Ser450Leu mutation encoded within the *rpoB* gene. An Ala90Val mutation encoded within the *gyrA* gene, either on its own or in combination with a Ser91Pro mutation, was also associated with elevated MICs for fluoroquinolones (levofloxacin and moxifloxacin) on the UKMYC5 plate.

For isoniazid, we applied a more nuanced test, since one strain contained a mutation (C → T at position −15) in the promoter of *inhA* that is known to confer only a low level of resistance, whereas eight strains contained the common Ser315Thr mutation (either alone or in combination with other mutations) encoded within the *katG* gene, which is known to confer high-level isoniazid resistance. The promoter mutation was determined to be resistant by MGIT, and APM measured the MIC to be 0.4 mg/liter—just twice the critical concentration. Not only did the strains containing either of these mutations consistently have MICs higher than those for the remaining strains, but the strain with the mutation known to confer low-level resistance was also correctly identified on the UKMYC5 plate (Fig. 6B) (24).

This *inhA* promoter mutation is known to also confer resistance to ethionamide. A second strain contains an *ethA* mutation that is also associated with resistance to this compound. The resulting MIC distributions for ethionamide-resistant and -susceptible strains are notably cleaner and better separated than those obtained when MGIT was used to classify the strains. No strain contained mutations known to confer resistance to linezolid. Despite some knowledge of their resistance mechanisms, the MIC distributions of the strains identified as resistant to clofazimine and *para*-aminosalicylic acid overlap those of the strains assumed to be sensitive.

Interestingly, the EQA panel included two strains that are resistant to delamanid according to APM. These have mutations in either the *ddn* gene or the *fbiC* (Rv1173) gene. The *ddn* mutation was associated with higher MICs than those seen with the *fbiC* mutation (Fig. 6C; Table S18) on the UKMYC5 plate. Consistent with this mutation having a small effect, MGIT classified the strain as sensitive, and the MIC measured by APM was 0.25 mg/liter—8× lower than that for the strain with the *ddn* mutation. Finally, four strains have mutations in *embB* that are known to confer resistance to ethambutol (encoding Met306Ile, Met306Val, and Gln497Arg substitutions). The UKMYC5 MIC distributions for these strains are higher, on average, than the distributions observed for the remaining 15 strains.

## DISCUSSION

We have shown that it is optimal to read the UKMYC5 plate after 14 days of incubation by using the Thermo Fisher Sensititre Vizion digital MIC viewing system. Although the laboratory scientists were unable to read as many plates by using a mirrored box, this reading method achieved levels of reproducibility indistinguishable from those for the Vizion system and is less expensive so may be the preferred solution in low-income settings. The Vizion system has the added advantage of capturing a photograph of the M. tuberculosis growth on the UKMYC5 plate at the time of reading, which not only can form a valuable audit trail but also can be analyzed by software, providing an additional set of readings.

Using the Vizion system, 93.0% of UKMYC5 plates were readable after 14 days of incubation (Fig. 2). The agreements between two readers reading the same plate and reading two plates containing the same strain taken from different subcultures were 97.9% and 92.8%, respectively. The intra- and interlaboratory reproducibilities were 95.6% and 93.1%, respectively. Removing strains that had potential off-scale MICs did not significantly alter these values. Considering all 14 anti-TB compounds separately, we found that *para*-aminosalicylic acid (PAS) performed poorly, consistent with a previous study (13), and the other 13 compounds individually had interreader agreements of ≥97.7%, with intra- and interlaboratory reproducibilities of ≥93.4% and ≥87.1%, respectively (Fig. 3).

Since there are no established standards for drug susceptibility testing (DST) of M. tuberculosis, there are no thresholds defining an acceptable level of reproducibility. To permit us to make some inferences, we cautiously apply the standards for aerobic bacteria, although we emphasize that any conclusions we make about the reproducibility of the UKMYC5 plate will be tentative, since they will need to be revisited when a mycobacterial DST standard is established. For aerobic bacteria, a reproducibility of ≥95% is required for clinical use (25). Considering the plate as a whole, the intralaboratory reproducibility meets this standard after 14 days of incubation, but the interlaboratory reproducibility does not. Examining the drugs individually, we find that the MICs of PAS are not reproducible after 14 days of incubation, with intra- and interlaboratory values of 74.2% and 67.5%, respectively. Seven compounds do, however, satisfy the borrowed standard, having both intra- and interlaboratory reproducibilities of ≥95% (amikacin, ethambutol, ethionamide, isoniazid, kanamycin, levofloxacin, and rifampin), and with the exception of rifampin, considering only on-scale MICs does not reduce either value to a level below the threshold. Of the remaining six compounds, four (clofazimine, delamanid, moxifloxacin, and rifabutin) have intralaboratory reproducibilities of ≥95% but interlaboratory reproducibilities in the range of 90 to 95%, linezolid has both reproducibilities in the range of 90 to 95%, and bedaquiline has intra- and interlaboratory reproducibilities of 94.3% and 87.1%, respectively.

Assessing accuracy is more difficult, since there are no critical concentrations for the UKMYC5 plate or any other microtiter plate, and we thus assumed shared breakpoints with the comparator methods, as done previously (13, 14). That said, we have demonstrated that the vast majority of UKMYC5 MICs for the H37Rv reference strain for each drug are within one doubling dilution of at least one, and often all three, MICs (Fig. 5) measured by APM, resazurin microtiter assay (REMA), and three studies that used a frozen-form microtiter plate (15, 16, 19). If all 19 EQA strains are considered, we find that the UKMYC5 plate consistently and repeatedly measures elevated MICs for strains classified as resistant by APM or MGIT (Fig. 5). Finally, if we instead classify the strains according to whether the genome contains mutations known to confer resistance to a specific compound, we find that the UKMYC5 plate typically records elevated MICs for these strains, although we note that this analysis also makes clear our relative lack of knowledge of genetic resistance mechanisms for some compounds (notably clofazimine and PAS). Two strains contained mutations known to confer low levels of resistance to isoniazid and delamanid, and these strains recorded low MICs on the UKMYC5 plate that were distinct from those for the larger susceptible and resistant populations.

Broth-based microdilution methods for TB DST that either directly (8) or indirectly (5, 10, 11) measure mycobacterial growth at a range of drug concentrations, potentially enabling the determination of MIC values, have been proposed in the past. In 2011, the WHO examined whether noncommercial microdilution assays would be suitable for screening patients at risk of MDR-TB and conditionally recommended their use (26). Subsequent studies assessed their performance (12–14) and established MIC distributions for the H37Rv reference strain (15, 16), demonstrating a good correlation with endorsed methods.

This study moves the field forward: UKMYC5 is the first microtiter plate assay to incorporate both new (delamanid and bedaquiline) and repurposed (linezolid and clofazimine) drugs in a dry-well format that is convenient to transport and store. The results presented here indicate that the UKMYC5 plate is sufficiently reproducible for large-scale research susceptibility testing of Mycobacterium tuberculosis, and in particular, it appears to be able to detect genetic variants which confer low levels of resistance, which is an important subsidiary goal of the CRyPTIC Consortium. In future work, we shall assess the ability of the AMyGDA software to add some quality control and, we hope, to improve the reproducibility and accuracy of the measured MICs.

In its present form, the UKMYC5 plate is not yet suitable for clinical use. The overall reproducibility and accuracy of the plate would benefit from a redesign to remove PAS and to use the vacated wells to expand the range of concentrations for drugs whose measurements were most frequently reported at the extremes of the dilution range; this redesign is under way. We hope that by the time the CRyPTIC project has built a large data set for unbiased clinical samples using the UKMYC5 plate (and its successor), an antimicrobial susceptibility testing standard for mycobacteria will have been established, which will allow a formal evaluation of the suitability of broth-based microtiter plates for clinical use in diagnosing and treating tuberculosis.

The UKMYC5 plate does have several limitations that need to be acknowledged; these include the need for a preculture step, entailing a delay of up to 6 weeks before the plate is inoculated. This currently prevents the rapid turnaround of results, but we expect that this time will be reduced significantly by further development work to define the optimal inoculum from MGIT cultures. In addition, as with all liquid-based DST, a microtiter plate such as the UKMYC5 plate can be used only in a biosafety level 3 laboratory, which may affect its potential utility in low-resource settings. Despite these shortcomings, the possibility of a single microtiter plate that can provide MICs for a range of anti-TB compounds at relatively low cost should be pursued vigorously.

The determination of MIC values for a range of drugs allows therapeutic decisions to be more nuanced, since they can be guided by the degree of resistance to a drug and an understanding of the tolerability of drug doses required to overcome it. Unlike those with CC-based DST methods, in which all errors are categorical in nature, MIC errors can be marginal and thereby may be potentially less disruptive to treatment decisions. In addition to these advantages, the UKMYC5 plate assays 14 drugs at once, at low cost, giving a clear advantage over MGIT and APM. Including both new and repurposed drugs is a clear advantage over other microtiter plate-based quantitative DST methods.

Because different countries have different settings, it would be optimal if one could modify the design of the microtiter plate to reflect the drugs that are locally available or the makeup of locally recommended regimens. It is therefore key that while the ranges of MICs are standardized the combination of drugs included on the plate can be adjusted according to need without significantly increasing the manufacturing cost. A challenge to regulators is therefore whether the performance of the plate can be accredited for a menu of individual drugs from which country-specific plates can be constructed, rather than for a fixed plate layout.

Since the increasing global numbers of MDR-TB cases are a major obstacle to TB control, let alone its elimination, there is a growing need for a clinical assay that can provide quantitative data on the second-line, new, and repurposed drugs that clinicians will have to prescribe with increasing frequency in the future. The UKMYC5 plate and its successor have the potential to guide the appropriate treatment of MDR-TB directly through implementation in clinical microbiology laboratories fulfilling all biosafety requirements for handling M. tuberculosis complex (MTBC) isolates or by providing detailed and timely surveillance of the prevalence of different strains by country. The Foundation for Innovative and New Diagnostics (FIND) has already expressed interest in pursuing WHO endorsement of the UKMYC5 plate. In parallel, the impact of the plate on our understanding of the effects of genomic mutations on the MICs of various drugs is likely to inform the WHO's planned assessment of WGS-based DST in 2018 (1). Whether through direct measurement of MICs or predictions of MIC from genomic data, a new era of quantitative TB DST may be almost with us.

## MATERIALS AND METHODS

### Participating laboratories.

Thirty-one vials containing 19 external quality assessment (EQA) TB strains were distributed by the WHO Supranational Reference Laboratory at San Raffaele Scientific Institute (SRL), Milan, Italy, to six other participating laboratories (Fig. 1A). These were located in Germany (Institute of Microbiology and Laboratory Medicine, IML Red GmbH, Gauting), United Kingdom (Public Health England Regional Centre for Mycobacteriology, Birmingham), India (P. D. Hinduja National Hospital and Medical Research Centre and Foundation for Medical Research, Mumbai), South Africa (Centre for Tuberculosis at the National Institute for Communicable Diseases, Johannesburg), Peru (Mycobacterial Laboratory, Cayetano Heredía University, Lima), and Vietnam (Oxford University Clinical Research Unit in Vietnam, Ho Chi Minh City).

### M. tuberculosis strains.

Each participating laboratory received up to 31 culture vials of Mycobacterium tuberculosis (Fig. 1B; see Table S17 in the supplemental material). One contained the Mycobacterium tuberculosis H37Rv reference strain ATCC 27294, purchased from BEI Resources (Manassas, VA) (GenBank accession number AL123456) (27), and was labeled as such. Eleven EQA strains were sent in duplicate (one of which was also H37Rv) by the SRL, along with another eight additional unique EQA strains, bringing the total to 31 culture vials containing 19 distinct strains. Except for H37Rv ATCC 27294, other samples were coded CRY-1 to CRY-30 and therefore were used blindly (Fig. 1B).

H37Rv ATCC 27294 was chosen as the reference strain because it is susceptible to all antitubercular drugs on the UKMYC5 plate. The other 18 strains were chosen from clinical isolates or proficiency testing isolates stored in the repository at the SRL based on the presence of mutations known to confer resistance to specific drugs with a high degree of confidence (Table S16) and therefore could reasonably be expected to have intermediate or high MIC values on the UKMYC5 plate.

### Preparation of replicates.

Upon arrival, all strains were subcultured onto solid media as follows: H37Rv was subcultured in 10 different Lowenstein-Jensen tubes or 7H10 agar plates, while the remaining 30 strains of the validation panel were subcultured in duplicate onto solid medium (Lowenstein-Jensen or 7H10 agar plates). Therefore, each participating laboratory tested up to 70 UKMYC5 plates (Fig. 1C; Fig. S5).

### UKMYC5 design.

The UKMYC5 plate was designed by the CRyPTIC Consortium and manufactured by Thermo Fisher Scientific Inc., United Kingdom. Fourteen anti-TB drugs (rifampin, rifabutin, isoniazid, ethambutol, levofloxacin, moxifloxacin, amikacin, kanamycin, ethionamide, clofazimine, *para*-aminosalicylic acid, linezolid, delamanid, and bedaquiline) were included, at 5 to 8 doubling dilutions (Fig. S1; Table S1). Janssen Pharmaceutica and Otsuka Pharmaceutical Co., Ltd., provided bedaquiline and delamanid pure substances, respectively. Although pyrazinamide-only plates containing lyophilized substance in different stocks were tested, poor performance due to suboptimal broth pH conditions resulted in pyrazinamide being excluded from the UKMYC5 plate. Two batches of UKMYC5 plates were manufactured and distributed for this study.

### Inoculation protocol.

Scientists from all laboratories received training in plate inoculation and reading at the SRL. The standard operating procedure involved preparing a suspension at a 0.5 McFarland standard in saline Tween with glass beads (Thermo Fisher, Scientific Inc., USA) from 20- to 25-day-old colonies (or no later than 14 days after visible growth) grown on Lowenstein-Jensen or 7H10 agar medium. Duplicates were tested on different days and starting from different subcultures, thus also starting from different bacterial suspensions.

Suspensions were then diluted 100-fold by the addition of 100 μl of suspension to 10 ml of enriched 7H9 broth. Aliquots of 100 μl of standard inoculum (1.5 × 10^5^ CFU/ml; approximate range, 5 × 10^4^ to 5 × 10^5^ CFU/ml) were dispensed into wells by use of a semiautomated Sensititre Autoinoculator (Thermo Fisher, Scientific Inc., USA). Each plate was then sealed using a manufacturer-supplied rectangle of transparent plastic that remained in place throughout the 21-day incubation period.

### Measurement of MICs.

In each laboratory, two scientists independently read each microtiter plate, using three different methods (Thermo Fisher Sensititre Vizion digital MIC viewing system, mirrored box, and inverted-light microscope), at 7, 10, 14, and 21 days postinoculation (Fig. 1D). A plate was considered valid if both positive-control wells (containing no drug) showed acceptable and visible growth. MIC results and additional data were recorded locally on paper and in a shared Web-enabled database (Table S17). An image of each plate was captured using the Vizion system and was stored and subsequently analyzed by AMyGDA (automated mycobacterial growth detection algorithm) software (18). AMyGDA analysis was performed at the University of Oxford, using default settings (Fig. S2).

### Laboratory validation.

For site F, the proportion of readable results was anomalously high (≥94.2%), regardless of reading day (Fig. S6; Table S20), yet site F had an anomalously low intralaboratory reproducibility, with values ranging from 77.5% to 80.2% depending on the reading day (Fig. S6; Table S20). Site F also had an anomalously low interlaboratory reproducibility of only 72.2% to 75.3% (Fig. S6; Table S18). Logistic mixed-effects models confirmed that, for site F, interreader agreement (results being within a doubling dilution) and intra- and interlaboratory reproducibilities were all significantly lower than those for the other laboratories (*P* < 0.001 for all comparisons) (Table S19). Data from site F were consequently excluded from subsequent analyses. A retrospective analysis of the plate images recorded by the Vizion system revealed that readers at site F had frequently mistaken sediment from the inoculum for bacterial growth, thereby allowing us to offer targeted training to the laboratory scientists involved. This process highlights the importance of storing images of all the plates to form an audit trail.

### Independent characterization of panel strains.

Each of the 19 strains used (Fig. 1B) was phenotypically characterized by the Bactec MGIT960 method (BD Lifesciences, NJ, USA), the Middlebrook 7H10/7H11 agar dilution method (Table S20), and (with the exception of ethionamide and *para*-aminosalicylic acid) the resazurin microtiter assay (REMA) (Table S21) for drugs for which the WHO has endorsed critical concentrations (CCs) (23). Middlebrook 7H11 agar was used for bedaquiline and delamanid. All strains were also subjected to whole-genome sequencing (WGS). Genomic DNA was extracted from Lowenstein-Jensen cultures by use of either FastPrep24 for cell lysis and ethanol precipitation or the cetyltrimethylammonium bromide method, as described elsewhere (28). Paired-end libraries of 101 bp were prepared using a Nextera XT DNA sample preparation kit (Illumina Inc., San Diego, CA, USA) and were sequenced on Illumina HiSeq 2500 instruments (with the exception of one site which used NextSeq 500 instruments). A minimum genome coverage of 30× was required for single nucleotide polymorphism (SNP) analysis. Variant calling for genes associated with resistance was performed by use of the PhyResSE Web tool and the bioinformatics pipeline at the SRL (29), and the results are given in Tables S17 and S18.

### Statistical analysis.

Both descriptive and modeling analyses were conducted. For the latter, logistic mixed-effects models were constructed, since the data consisted of repeated measurements. A drug on a plate was defined as readable if (i) there was acceptable growth in both positive-control wells, (ii) there was no contamination in the wells for that drug, and (iii) the contents of the wells for that drug had not evaporated. We define two measurements as being in agreement if the two MICs are within one doubling dilution of each other. Furthermore, reproducibility is defined as the proportion of MICs that are within one doubling dilution of the mode. Due to the high percentage of off-scale results, results were considered to be within one doubling dilution if they were adjacent in the doubling dilution range, even if one of them was off-scale. This constitutes a best-case approach, and therefore, where appropriate, calculations were repeated using only drug-strain combinations for which the MIC mode was on-scale. To assess the intralaboratory reproducibility, the modal MIC for each drug was computed for that site, with pooling of the results across reading methods, days, replicates, and readers. To test the interlaboratory reproducibility, the mode was calculated only for each drug, with pooling of the results across sites as well as across reading methods, days, replicates, and readers. To assess the accuracy of the plate, we compared UKMYC5 MICs to the modes of the MICs obtained by other established DST methods. For both APM and REMA, two independent measurements were taken for each drug (with the exception of ethionamide and PAS, which were not evaluated), and if there was a discrepancy (i.e., the mode was not unique), the lower value was chosen. Since no critical concentrations exist for UKMYC5 plates, we used the same critical concentrations as those for the comparator method (as done previously [13, 14]). Results were assessed in the following three ways: (i) the agreement between the UKMYC5 plate results and those obtained by an established method was calculated; (ii) categorical agreement was defined as concordant reporting of either sensitivity or resistance, as defined by the critical concentration (CC) for the comparator phenotypic test (if the MIC was lower than or equal to the CC, the strain was defined as susceptible; otherwise it was defined as resistant); and (iii) conditional agreement was defined as resistance by the comparator method and a MIC equal to or higher than the CC on the UKMYC5 plate or susceptibility by the comparator method and a MIC equal to or lower than the CC plus one doubling dilution on the UKMYC5 plate (14).

### Accession number(s).

The sequences reported in this paper have been deposited in the Sequence Read Archive of the National Center for Biotechnology Information under study accession numbers SRP068011 and SRP130092.

## Supplementary Material

Supplemental file 1

Supplemental file 2
