# Supplementary material for: Validating a 14-Drug Microtiter Plate Containing Bedaquiline and Delamanid for Large-Scale Research Susceptibility Testing of Mycobacterium tuberculosis
Source: Antimicrob Agents Chemother. 2018 Aug 27;62(9):e00344-18. doi: 10.1128/AAC.00344-18 (PMC6125532; doi:10.1128/AAC.00344-18)
Supplement: Supplemental file 1 [file zac009187425s1.pdf]

Supplemental Material

SENSITITRE CUSTOM PLATE FORMAT

2203161639

Plate Code: **UKMYC5** Date: **6-Apr-16**

	1	2	3	4	5	6	7	8	9	10	11	12
A	BDQ 2	KAN 16	KAN 8	KAN 4	KAN 2	KAN 1	ETH 8	ETH 4	ETH 2	ETH 1	ETH 0.5	ETH 0.25
B	BDQ 1	AMI 8	EMB 8	INH 1.6	LEVO 8	MXF 4	DLM 1	LZD 2	CFZ 4	RIF 4	RFB 2	PAS 4
C	BDQ 0.5	AMI 4	EMB 4	INH 0.8	LEVO 4	MXF 2	DLM 0.5	LZD 1	CFZ 2	RIF 2	RFB 1	PAS 2
D	BDQ 0.25	AMI 2	EMB 2	INH 0.4	LEVO 2	MXF 1	DLM 0.25	LZD 0.5	CFZ 1	RIF 1	RFB 0.5	PAS 1
E	BDQ 0.12	AMI 1	EMB 1	INH 0.2	LEVO 1	MXF 0.5	DLM 0.12	LZD 0.25	CFZ 0.5	RIF 0.5	RFB 0.25	PAS 0.5
F	BDQ 0.06	AMI 0.5	EMB 0.5	INH 0.1	LEVO 0.5	MXF 0.25	DLM 0.06	LZD 0.12	CFZ 0.25	RIF 0.25	RFB 0.12	PAS 0.25
G	BDQ 0.03	AMI 0.25	EMB 0.25	INH 0.05	LEVO 0.25	MXF 0.12	DLM 0.03	LZD 0.06	CFZ 0.12	RIF 0.12	RFB 0.06	PAS 0.12
H	BDQ 0.015	EMB 0.06	EMB 0.12	INH 0.025	LEVO 0.12	MXF 0.06	DLM 0.015	LZD 0.03	CFZ 0.06	RIF 0.06	POS	POS

ANTIMICROBICS	
BDQ	Bedaquiline
DLM	Delamanid
AMI	Amikacin
CFZ	Clofazimine
EMB	Ethambutol
ETH	Ethionamide
INH	Isoniazid
KAN	Kanamycin
LEVO	Levofloxacin
LZD	Linezolid
MXF	Moxifloxacin
PAS	Para-aminosalicylic acid
POS	Positive Control
RFB	Rifabutin
RIF	Rifampin

Figure S1. The layout of the UKMYC5 microtitre plate. Note that the two wells marked POS in the bottom right contain no antibiotic and are therefore positive controls. Growth must be observed in both wells for a plate to be read.

Table S1. The concentration ranges of the 14 drugs present on the UKMYC5 plate. As shown in Figure S1, these all form doubling dilutions.

Drug name	Drug abbreviation	Number of wells	Testing Range (mg/L)
Rifampicin	RIF	7	0.06 - 4
Rifabutin	RFB	6	0.06 - 2
Ethambutol	EMB	8	0.06 - 8
Isoniazid	INH	7	0.02 – 1.6
Kanamycin	KAN	5	1 - 16
Amikacin	AMI	6	0.25 - 8
Levofloxacin	LEV	7	0.125 - 8
Moxifloxacin	MXF	7	0.06 - 4
Clofazimine	CFZ	7	0.06 - 4
Linezolid	LZD	7	0.03 - 2
Para-aminosalicylic acid	PAS	6	0.125 - 4
Ethionamide	ETH	6	0.25 - 8
Bedaquiline	BDQ	8	0.015 - 2
Delamanid	DLM	7	0.015 - 1

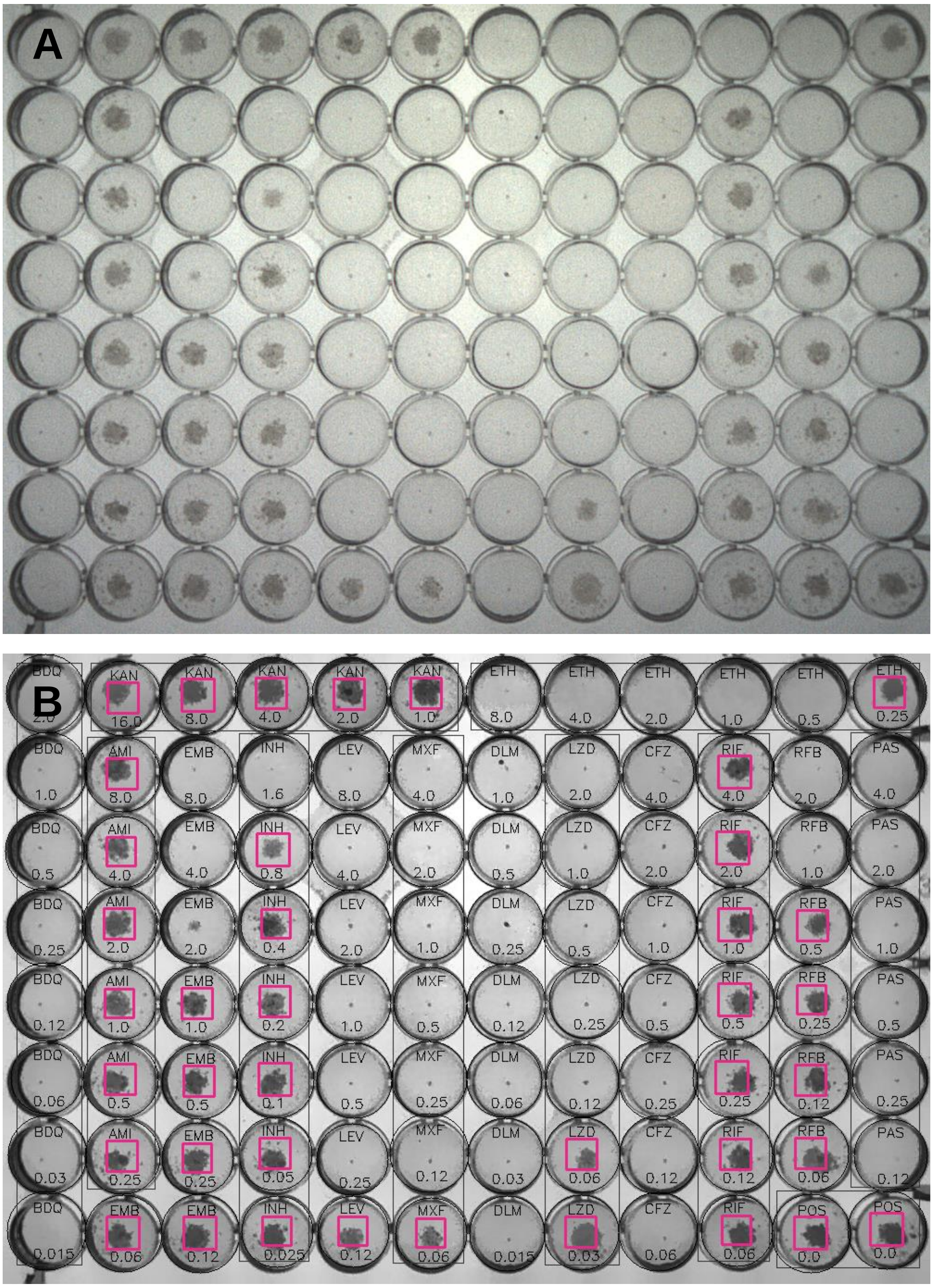

Figure S2. An image of a UKMYC5 plate processed by the AMyGDA software. (**A**) the raw bitmap image recorded by the Vizion™ Digital Viewing system. (**B**) The AMyGDA software filters the image to improve the contrast and homogenise the illumination before identifying the location of all 96-wells, annotated and drug and concentration and finally measuring the amount of growth in each well. Wells which the software determines contain growth are marked with a pink square. This image was taken 14 days after inoculation.

Table S2. Related to Figure 2. For each reading method, (**A**) the percentage of readable results, (**B**) the inter-reader agreement, (**C**) the agreement between duplicated strains and the (**D**) intra- (**E**) inter-laboratory reproducibility. All values include both off- and on-scale measurements. Site F excluded.

(A) Percentage (%) of readable results

Reading Method	Day 7	Day 10	Day 14	Day 21
Vizion	60.2	79.6	93.0	97.6
Mirrored-box	57.8	75.6	85.7	95.9
Microscope	66.1	77.8	87.4	96.0

(**B**) Inter-reader agreement (%)

Reading Method	Day 7	Day 10	Day 14	Day 21
Vizion	95.5	96.5	97.9	98.0
Mirrored-box	96.6	96.2	97.8	98.2
Microscope	92.7	93.5	96.5	96.8

(**C**) Agreement between duplicated strains (%)

Reading Method	Day 7	Day 10	Day 14	Day 21
Vizion	89.7	90.6	92.8	92.9
Mirrored-box	91.9	91.1	92.9	92.5
Microscope	89.1	89.7	91.2	90.7

(**D**) Intra-laboratory reproducibility (%)

Reading Method	Day 7	Day 10	Day 14	Day 21
Vizion	90.2	94.4	95.6	92.2
Mirrored-box	91.9	94.8	95.9	92.2
Microscope	91.1	92.9	92.9	88.1

(**E**) Inter-laboratory reproducibility (%)

Reading Method	Day 7	Day 10	Day 14	Day 21
Vizion	89.9	92.8	93.1	89.4
Mirrored-box	92.0	92.8	92.7	89.2
Microscope	90.9	91.0	89.9	85.4

Table S3. For each reading method, the (**A**) the agreement between duplicated strains and the (**B**) intra- (**C**) inter-laboratory reproducibility. These analyses include for each drug, only the data of the strains with the MIC mode on-scale. Site F excluded.

(A) Inter-reader agreement (%)

Reading Method	Day 7	Day 10	Day 14	Day 21
Vizion	95.7	97.4	98.5	98.7
Mirrored-box	96.8	97.0	98.7	99.0
Microscope	93.0	95.2	97.7	97.9

(**B**) Intra-laboratory reproducibility (%)

Reading Method	Day 7	Day 10	Day 14	Day 21
Vizion	89.1	94.6	96.5	94.6
Mirrored-box	91.3	94.8	96.9	94.6
Microscope	91.7	94.5	95.1	90.7

(**C**) Inter-laboratory reproducibility (%)

Reading Method	Day 7	Day 10	Day 14	Day 21
Vizion	87.3	92.6	94.9	93.7
Mirrored-box	89.9	92.9	94.7	93.6
Microscope	90.8	92.8	93.1	90.4

Table S4. Related to Figure 2. The percentage of measurements that were off-scale for each drug, by reading method and reading day. The drugs with the smallest proportion of off-scale MICs measured using the UKMYC5 plate were EMB, LEV, LZD and MXF. Site F excluded.

	VIZION				MIRROR				MICROSCOPE			
	**day7**	**day10**	**day14**	**day21**	**day7**	**day10**	**day14**	**day21**	**day7**	**day10**	**day14**	**day21**
**AMK**	87.2	78.5	68.1	53.9	83.5	74.7	66.2	53.9	66.1	63.8	59.2	48.6
**BDQ**	52.6	40.6	26.1	11.8	47.3	35.9	23.2	11.1	40.9	31.3	21.1	10.8
**CFZ**	66.2	66.1	61.6	48.0	67.7	62.6	59.2	45.5	56.4	54.8	50.7	39.0
**DLM**	91.4	86.7	79.9	57.4	86.9	84.1	77.3	55.6	83.9	81.4	71.9	50.1
**EMB**	6.1	5.1	5.0	8.0	4.1	4.2	4.2	7.4	5.0	4.4	5.6	8.9
**ETH**	12.6	10.4	10.4	11.8	10.6	9.4	10.7	11.9	11.8	10.2	11.3	13.1
**INH**	51.8	45.4	40.4	38.6	46.9	41.4	36.9	37.9	44.1	40.6	34.2	38.6
**KAN**	74.5	56.9	46.5	40.3	74.2	53.7	45.4	41.2	51.9	45.1	40.0	36.7
**LEV**	10.8	8.7	6.1	6.6	7.3	6.5	6.0	6.9	8.4	6.8	6.8	7.3
**LZD**	3.5	1.7	0.7	0.5	1.4	1.2	0.9	0.8	0.6	1.2	0.9	0.4
**MXF**	15.5	10.0	8.1	7.4	11.7	7.5	7.6	7.2	10.0	7.2	8.4	7.8
**PAS**	82.5	66.4	43.2	30.0	83.1	60.5	43.5	30.1	57.8	44.4	32.9	25.6
**RFB**	77.4	80.6	80.5	81.4	79.0	79.1	81.1	82.4	75.4	79.8	81.9	83.2
**RIF**	83.9	77.5	72.2	57.6	80.4	74.7	69.9	56.3	72.6	71.4	63.0	49.0

Table S5. Logistic mixed-effects models for readable results, inter-reader agreement and agreement between duplicated strains (excluding Site F, adjusted for drug, interaction between reading day and drug). Random effects were defined nested with respect to strains, sites and replicates.

	**Readable results**			**Inter-reader agreement**			**Agreement between duplicated strains**		
**Parameter**	**Estimate**	**SE**	**p-value**	**Estimate**	**SE**	**p-value**	**Estimate**	**SE**	**p-value**
Intercept	4.939	0.336	**<0.001**	4.926	0.297	**<0.001**	3.805	0.296	**<0.001**
READINGDAY									
Day 7 vs 14	-3.846	0.119	**<0.001**	-1.246	0.335	**<0.001**	-1.482	0.324	**<0.001**
Day 10 vs 14	-1.993	0.119	**<0.001**	-0.609	0.337	0.071	-0.633	0.326	0.052
Day 21 vs 14	1.816	0.187	**<0.001**	-0.103	0.355	0.772	-0.414	0.313	0.185
METHOD									
MIRROR vs VIZION	-1.236	0.059	**<0.001**	-0.154	0.157	0.327	-0.031	0.123	0.803
MICROSCOPE vs VIZION	-1.007	0.060	**<0.001**	-0.692	0.141	**<0.001**	-0.300	0.116	**0.010**
Day 7: MIRROR	0.988	0.073	**<0.001**	0.501	0.215	**0.020**	0.247	0.183	0.178
Day 10:MIRROR	0.835	0.073	**<0.001**	0.111	0.206	0.590	0.152	0.169	0.368
Day 21:MIRROR	0.489	0.111	**<0.001**	0.245	0.220	0.267	-0.035	0.169	0.834
Day 7: MICROSCOPE	1.567	0.073	**<0.001**	0.380	0.186	**0.042**	0.459	0.168	**0.006**
Day 10:MICROSCOPE	0.820	0.074	**<0.001**	-0.0002	0.185	0.999	0.262	0.161	0.104
Day 21: MICROSCOPE	0.277	0.112	0.0130	0.108	0.197	0.582	-0.026	0.161	0.873

Table S6. Logistic mixed-effects models for intra- and inter-laboratory reproducibility (excluding Site F, adjusted for drug, interaction between reading day and drug). Random effects were defined nested with respect to strains, sites and replicates.

	**Intra-laboratory reproducibility**			**Inter-laboratory reproducibility**		
**Parameter**	**Estimate**	**SE**	**p-value**	**Estimate**	**SE**	**p-value**
Intercept	3.918	0.156	**<0.001**	3.541	0.145	**<0.001**
READINGDAY						
Day 7 vs 14	-1.059	0.183	**<0.001**	-0.904	0.171	**<0.001**
Day 10 vs 14	-0.319	0.191	0.096	-0.143	0.180	0.426
Day 21 vs 14	-1.001	0.162	**<0.001**	-0.954	0.147	**<0.001**
METHOD						
MIRROR vs VIZION	0.051	0.077	0.505	-0.079	0.062	0.199
MICROSCOPE vs VIZION	-0.615	0.068	**<0.001**	-0.488	0.058	**<0.001**
Day 7: MIRROR	0.190	0.102	0.063	0.344	0.090	**<0.001**
Day 10:MIRROR	0.049	0.106	0.641	0.075	0.089	0.399
Day 21:MIRROR	-0.068	0.096	0.475	0.043	0.080	0.593
Day 7: MICROSCOPE	0.828	0.094	**<0.001**	0.655	0.085	**<0.001**
Day 10:MICROSCOPE	0.354	0.096	**<0.001**	0.224	0.084	**0.008**
Day 21: MICROSCOPE	0.027	0.087	0.752	0.007	0.076	0.928

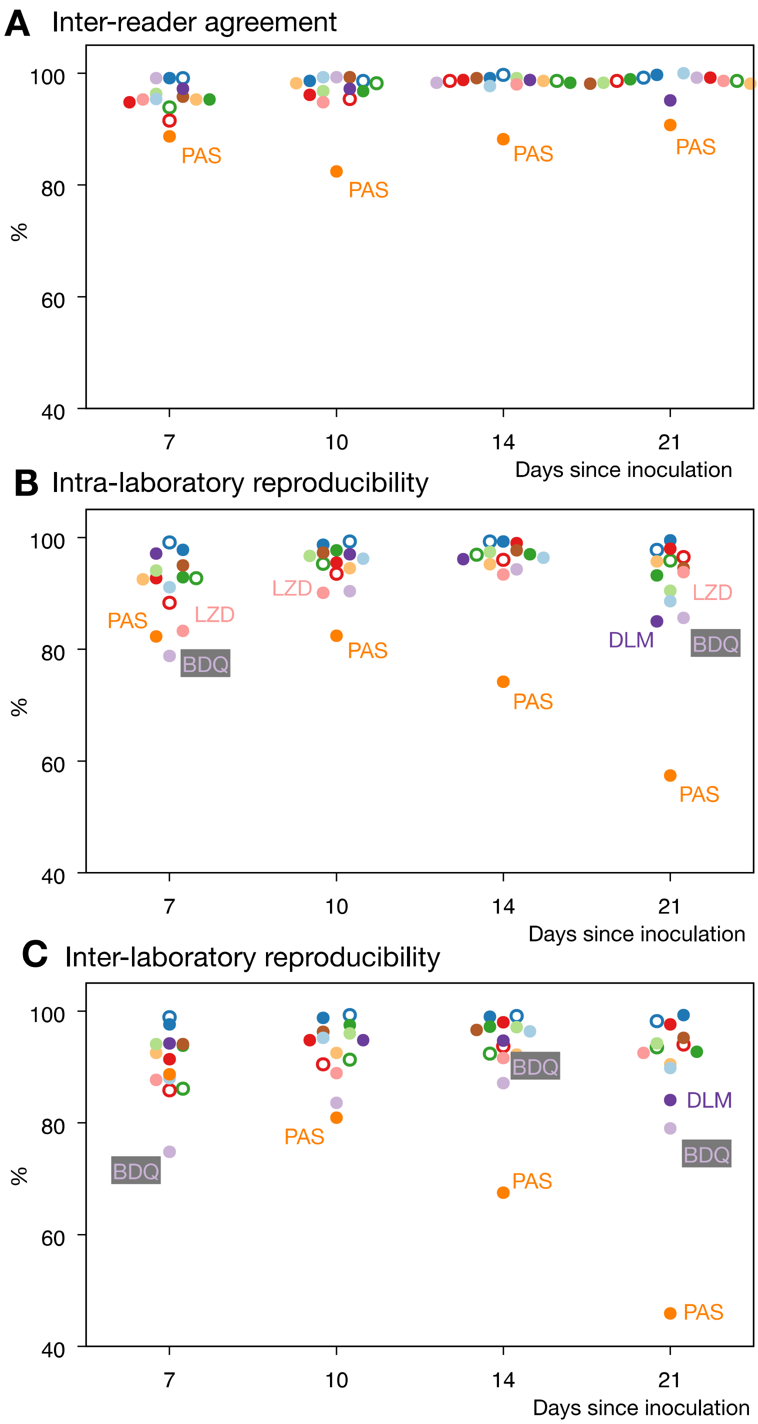

Figure S3. Related to Figure 3. The (**A**) inter-reader agreement and (**B**) intra- and (**C**) inter-laboratory reproducibility for each drug across all reading days (only Vizion measurements, excluding Site F). PAS shows the lowest agreement between readers at each reading day. The drugs are labelled using the abbreviations defined in Table S1 and coloured according to the scheme in Figure 3.

Table S7. Related to Figure 3. The inter-reader agreement and intra- and inter-laboratory reproducibilities for each drug (only Vizion, excluding Site F). PAS is either the lowest value, or is in the lowest values of all three metrics, across all reading days.

Drug	Inter-reader agreement (%)				Intra-laboratory reproducibility (%)				Inter-laboratory reproducibility (%)			
	**Day 7**	**Day10**	**Day14**	**Day21**	**Day7**	**Day10**	**Day14**	**Day21**	**Day7**	**Day10**	**Day14**	**Day21**
AMK	99.1	98.6	99.1	99.7	97.8	98.7	99.3	99.5	97.6	98.8	99.0	99.3
BDQ	99.1	99.3	98.3	99.2	78.8	90.4	94.3	85.6	74.8	83.6	87.1	79.0
CFZ	95.3	98.2	98.6	98.1	92.5	94.5	95.2	95.7	92.5	92.5	92.2	90.5
DLM	97.2	97.2	98.8	95.1	97.1	97.0	96.1	85.0	94.2	94.8	94.7	84.1
EMB	95.4	99.3	97.7	100.0	91.1	96.2	96.4	88.6	88.0	95.2	96.4	89.8
ETH	96.3	96.8	99.1	98.3	94.1	96.7	97.4	90.5	94.1	96.0	97.1	94.2
INH	95.8	99.3	99.1	98.1	95.0	97.3	97.7	94.5	94.1	96.3	96.6	95.2
KAN	99.1	98.6	99.7	99.2	99.1	99.3	99.3	97.8	98.9	99.3	99.1	98.2
LEV	94.8	96.1	98.8	99.2	92.7	95.5	99.0	98.0	91.4	94.8	98.0	97.6
LZD	95.3	94.8	98.0	98.6	83.3	90.1	93.4	93.8	87.7	88.9	91.6	92.5
MOX	91.5	95.4	98.6	98.6	88.3	93.5	96.0	96.5	85.8	90.5	93.7	94.0
PAS	88.7	82.4	88.2	90.7	82.3	82.4	74.2	57.4	88.7	80.9	67.5	45.9
RFB	93.9	98.2	98.6	98.6	92.7	95.3	96.9	95.9	86.1	91.3	92.4	93.5
RIF	95.3	96.8	98.3	98.9	92.9	97.7	97.0	93.2	93.8	97.5	97.2	92.7

Table S8. The inter-reader agreement, intra- and inter-laboratory reproducibilities for each drug (only Vizion, excluding Site F) calculated only for (drug, strain) combinations where the mode MIC was 'on-scale' i.e. was neither the first nor last well.

Drug	n. strains with mode on-scale	Inter-reader agreement (%)				Intra-laboratory reproducibility (%)				Inter-laboratory reproducibility (%)			
		**Day 7**	**Day10**	**Day14**	**Day21**	**Day7**	**Day10**	**Day14**	**Day21**	**Day7**	**Day10**	**Day14**	**Day21**
AMK	4	100	99.1	100	100	100	99.6	100	98.9	100	100	100	100
BDQ	8	99.2	100	97.9	99.5	68.9	87	94.6	91.2	59.6	77	89.8	94.6
CFZ	3	94.7	95.3	98.1	96.5	83.8	85	86.9	90.6	82.5	83.9	83.2	83.8
DLM	1	83.3	78.6	93.3	88.9	84	89.7	96.8	91.7	40	51.7	71	86.1
EMB	18	95	99.3	97.9	100	90.5	96	96.4	88	87.9	94.9	96.7	89.5
ETH	17	96.2	97.6	99	98.2	95	97.6	97.3	89.5	95.2	96.8	97	93.6
INH	10	96.1	99.4	99	96.8	96.2	98.3	97.6	92.9	95.5	97.5	96.7	93.6
KAN	8	100	100	100	98.9	100	99.7	98.9	96	100	100	99.4	98.9
LEV	18	94.4	95.9	98.8	99.1	92.2	95.2	99	97.9	90.8	94.5	97.9	97.4
LZD	19	95.3	94.8	98	98.6	83.3	90	93.5	93.8	87.7	88.9	91.6	92.5
MXF	18	90.9	95.2	98.5	98.6	87.4	93.1	95.8	96.3	85.3	90	93.4	93.7
PAS	0	-	-	-	-	-	-	-	-	-	-	-	-
RFB	1	100	100	94.7	100	100	93.3	92.1	86.5	61.5	80	84.2	89.2
RIF	4	100	100	96.7	98.4	84.8	97.1	91.9	84.9	91.1	96.2	92.7	85.7
All		95.7	97.4	98.5	98.7	89.4	94.6	96.3	93.2	87.9	92.5	94.9	93.7

Table S9. Logistic mixed-effects models for inter-reader agreement and intra- and inter-laboratory reproducibilities (only Vizion, excluding Site F). Random effects were defined nested with respect to strains, sites and replicates.

	**Inter-reader agreement**			**Intra-laboratory reproducibility**			**Inter-laboratory reproducibility**		
	**Estimate**	**SE**	**p-value**	**Estimate**	**SE**	**p-value**	**Estimate**	**SE**	**p-value**
Intercept	4.708	0.444	**<0.001**	3.753	0.235	**<0.001**	3.620	0.242	**<0.001**
READINGDAY									
Day 7 vs 14	-1.177	0.542	**0.030**	-0.949	0.294	**0.001**	-0.884	0.303	**0.004**
Day 10 vs 14	-0.673	0.548	0.219	0.259	0.354	0.465	0.096	0.349	0.784
Day 21 vs 14	0.453	0.661	0.493	-0.908	0.270	**0.001**	-0.988	0.271	**<0.001**
Drug									
AMK vs RIF	0.725	0.722	0.316	1.482	0.504	**0.003**	1.079	0.445	**0.015**
BDQ vs RIF	-0.003	0.595	0.995	-0.704	0.280	**0.012**	-1.674	0.257	**<0.001**
CFZ vs RIF	0.193	0.623	0.757	-0.518	0.287	0.071	-1.094	0.270	**<0.001**
DLM vs RIF	0.425	0.662	0.521	-0.272	0.301	0.366	-0.659	0.286	**0.021**
EMB vs RIF	-0.308	0.559	0.581	-0.186	0.305	0.543	-0.235	0.308	0.445
ETH vs RIF	0.723	0.722	0.317	0.160	0.330	0.627	-0.002	0.324	0.994
INH vs RIF	0.724	0.722	0.316	0.269	0.340	0.430	-0.199	0.311	0.522
KAN vs RIF	1.845	1.090	0.091	1.482	0.504	**0.003**	1.237	0.471	**0.009**
LEV vs RIF	0.426	0.662	0.520	1.139	0.443	**0.010**	0.371	0.355	0.297
LZD vs RIF	-0.165	0.575	0.775	-0.853	0.274	**0.002**	-1.174	0.268	**<0.001**
MXF vs RIF	0.193	0.623	0.757	-0.307	0.298	0.303	-0.845	0.278	**0.002**
PAS vs RIF	-2.244	0.459	**<0.001**	-2.581	0.243	**<0.001**	-2.943	0.244	**<0.001**
RFB vs RIF	0.192	0.623	0.758	-0.050	0.314	0.874	-1.053	0.271	**<0.001**
Day 7:AMK	1.009	1.073	0.347	-0.218	0.627	0.728	-0.083	0.575	0.885
Day 10:AMK	0.142	0.951	0.881	-0.902	0.676	0.182	-0.296	0.642	0.645
Day 21:AMK	0.695	1.339	0.604	1.169	0.727	0.108	1.415	0.649	**0.029**
Day 7:BDQ	1.732	0.993	0.081	-0.648	0.358	0.070	-0.036	0.343	0.917
Day 10:BDQ	1.584	0.994	0.111	-0.837	0.417	**0.044**	-0.426	0.385	0.269
Day 21:BDQ	0.301	0.979	0.759	-0.192	0.336	0.568	0.389	0.309	0.208
Day 7:CFZ	-0.193	0.785	0.806	0.449	0.389	0.248	0.880	0.381	0.021
Day 10:CFZ	0.440	0.849	0.605	-0.400	0.437	0.360	-0.083	0.409	0.839
**Day 21:CFZ**	-0.781	0.895	0.383	1.011	0.373	**0.007**	0.797	0.332	**0.016**
Day 7:DLM	0.145	0.857	0.866	1.252	0.455	**0.006**	0.737	0.403	0.068
Day 10:DLM	-0.298	0.834	0.721	0.003	0.473	0.994	-0.113	0.431	0.793
Day 21:DLM	-2.033	0.876	**0.020**	-0.664	0.354	0.060	-0.244	0.338	0.470
Day 7:EMB	0.312	0.736	0.672	-0.073	0.396	0.853	-0.500	0.394	0.205
Day 10:EMB	1.893	0.973	0.052	-0.335	0.463	0.470	-0.451	0.448	0.315
Day 21:EMB	14.242	314.704	0.964	-0.418	0.361	0.247	-0.141	0.362	0.697
Day 7:ETH	-0.469	0.881	0.594	0.046	0.431	0.915	0.070	0.429	0.871
Day 10:ETH	-0.722	0.875	0.409	-0.529	0.487	0.277	-0.479	0.467	0.306
Day 21:ETH	-1.158	0.979	0.237	-0.541	0.385	0.161	0.269	0.390	0.490
Day 7:INH	-0.603	0.873	0.490	0.107	0.446	0.811	0.260	0.420	0.536
Day 10:INH	0.852	1.075	0.428	-0.416	0.506	0.412	-0.203	0.462	0.660
Day 21:INH	-1.322	0.967	0.172	-0.044	0.407	0.914	0.661	0.385	0.086
Day 7:KAN	-0.112	1.349	0.934	0.727	0.737	0.324	0.568	0.681	0.404
Day 10:KAN	-0.978	1.254	0.435	-0.195	0.763	0.799	0.114	0.737	0.877

Day 21:KAN	-1.547	1.339	0.248	-0.253	0.584	0.665	0.280	0.567	0.621
Day 7:LEV	-0.536	0.811	0.509	-1.173	0.516	0.023	-0.734	0.441	0.096
Day 10:LEV	-0.649	0.813	0.425	-1.837	0.558	0.001	-1.135	0.480	**0.018**
Day 21:LEV	-0.126	1.021	0.902	0.158	0.537	0.768	0.812	0.453	0.073
Day 7:LZD	0.166	0.748	0.825	-0.184	0.357	0.606	0.395	0.363	0.277
Day 10:LZD	-0.410	0.728	0.574	-0.739	0.411	0.073	-0.449	0.398	0.260
Day 21:LZD	-0.067	0.895	0.940	0.946	0.349	**0.007**	1.152	0.336	**0.001**
Day 7:MXF	-0.896	0.756	0.236	-0.285	0.384	0.457	-0.108	0.368	0.769
Day 10:MXF	-0.604	0.773	0.435	-0.796	0.439	0.069	-0.600	0.409	0.142
Day 21:MXF	-0.424	0.926	0.647	1.025	0.391	**0.009**	1.074	0.351	**0.002**
Day 7:PAS	1.174	0.616	0.057	1.469	0.333	**0.000**	2.262	0.349	**<0.001**
Day 10:PAS	0.109	0.602	0.856	0.289	0.382	0.450	0.651	0.375	0.082
Day 21:PAS	-0.165	0.713	0.816	0.040	0.296	0.893	0.022	0.294	0.941
Day 7:RFB	-0.498	0.770	0.518	0.012	0.411	0.977	0.118	0.363	0.744
Day 10:RFB	0.440	0.849	0.604	-0.688	0.461	0.135	-0.287	0.406	0.479
Day 21:RFB	-0.425	0.926	0.647	0.611	0.397	0.123	1.183	0.343	**0.001**

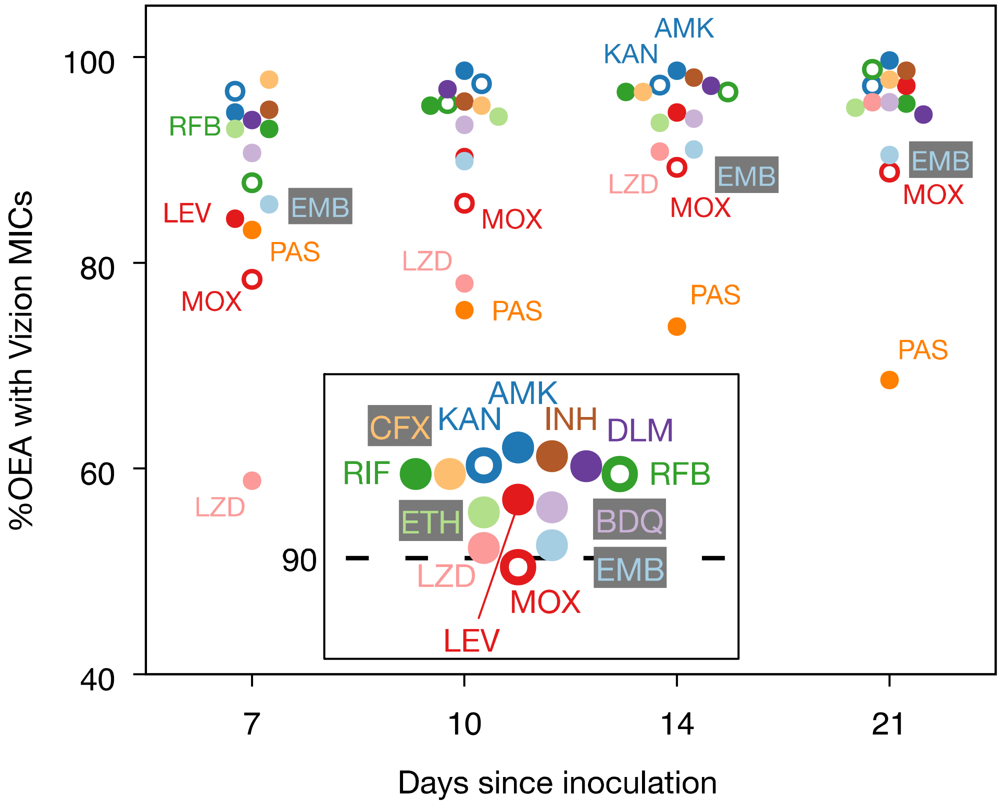

Fig S4. The proportion of results in agreement (OEA) between MICs read by Vizion™ and determined by the AMyGDA software (Table S9). Drugs are labelled using the abbreviations defined in Table S1.

Table S10. Related to Figure S3. The proportion of MICs read by Vizion™ in agreement (±1 doubling dilution) with those determined by the AMyGDA software.

	**Day 7**	**Day 10**	**Day 14**	**Day 21**
AMK	94.6	98.7	98.7	99.7
BDQ	90.7	93.4	94.0	95.6
CFZ	97.8	95.3	96.6	97.8
DLM	93.9	96.9	97.2	94.4
EMB	85.7	89.9	91.0	90.5
ETH	93.0	94.2	93.6	95.1
INH	94.9	95.7	98.0	98.7
KAN	96.7	97.4	97.3	97.2
LEV	84.3	90.3	94.6	97.2
LZD	58.8	78.0	90.8	95.6
MOX	78.4	85.8	89.3	88.8
PAS	83.2	75.4	73.8	68.6
RFB	87.8	95.5	96.6	98.8
RIF	93.0	95.3	96.6	95.5
Average	88.1	91.6	93.4	93.8

Table S11. Logistic mixed-effects models for the agreement between measurements taken using the Vizion™ reading system and the AMyGDA (automated Mycobacterial growth detection algorithm) software. Random effects were defined nested with respect to strains, sites and replicates.

	**Estimate**	**SE**	**p-value**
Intercept	3.574	0.357	**<0.001**
READINGDAY			
Day 7 vs 14	-0.696	0.475	0.143
Day 10 vs 14	-0.324	0.457	0.479
Day 21 vs 14	-0.275	0.433	0.525
Drug			
AMK vs RIF	1.056	0.605	0.081
BDQ vs RIF	-0.601	0.419	0.152
CFZ vs RIF	0.043	0.465	0.927
DLM vs RIF	0.282	0.492	0.566
**EMB vs RIF**	**-1.049**	**0.393**	**0.008**
ETH vs RIF	-0.646	0.411	0.116
INH vs RIF	0.534	0.532	0.316
KAN vs RIF	0.358	0.492	0.467
LEV vs RIF	-0.439	0.423	0.299
**LZD vs RIF**	**-1.048**	**0.391**	**0.007**
**MXF vs RIF**	**-1.234**	**0.384**	**0.001**
**PAS vs RIF**	**-2.465**	**0.360**	**<0.001**
RFB vs RIF	0.043	0.465	0.926
Day 7:AMK	-0.652	0.790	0.410
Day 10:AMK	0.399	0.900	0.657
Day 21:AMK	1.783	1.205	0.139
Day 7:BDQ	0.388	0.621	0.532
Day 10:BDQ	0.272	0.594	0.647
Day 21:BDQ	0.703	0.582	0.227
Day 7:CFZ	1.242	0.827	0.133
Day 10:CFZ	-0.014	0.646	0.982
**Day 21:CFZ**	0.805	0.667	0.228
Day 7:DLM	-0.023	0.709	0.974
Day 10:DLM	0.251	0.704	0.721
Day 21:DLM	-0.453	0.625	0.468
Day 7:EMB	0.189	0.578	0.743
Day 10:EMB	0.201	0.553	0.717
Day 21:EMB	0.246	0.523	0.639
Day 7:ETH	0.678	0.634	0.285
Day 10:ETH	0.463	0.591	0.433
Day 21:ETH	0.571	0.567	0.314
Day 7:INH	-0.105	0.749	0.889
Day 10:INH	-0.432	0.702	0.538
Day 21:INH	0.793	0.787	0.314
Day 7:KAN	0.513	0.758	0.499
Day 10:KAN	0.377	0.720	0.601
Day 21:KAN	0.201	0.662	0.762
Day 7:LEV	-0.467	0.596	0.433
Day 10:LEV	-0.356	0.576	0.537
Day 21:LEV	0.997	0.613	0.104
**Day 7:LZD**	**-1.408**	**0.551**	**0.011**
Day 10:LZD	-0.829	0.531	0.118
**Day 21:LZD**	**1.143**	**0.557**	**0.040**
Day 7:MXF	-0.126	0.557	0.822
Day 10:MXF	-0.011	0.534	0.984
Day 21:MXF	0.248	0.510	0.627
**Day 7:PAS**	**1.373**	**0.550**	**0.013**
Day 10:PAS	0.417	0.506	0.410
Day 21:PAS	-0.003	0.474	0.995
Day 7:RFB	-0.660	0.635	0.299
Day 10:RFB	0.049	0.645	0.940
Day 21:RFB	1.344	0.743	0.071

Table S12. Related to Figure 3. The agreement (between UKMYC5 MICs read by Vizion™ and frozen-form microtiter plate MICs.

	%agreement with the frozen-form microtitre plate mode MICs after:	
	**14 days**	**21 days**
AMK	100.0	100.0
BDQ	100.0	95.6
CFZ	96.8	95.6
EMB	98.7	83.0
INH	97.4	91.8
KAN	92.4	65.2
LEV	99.4	98.1
LZD	99.4	97.5
MXF	98.7	98.1
RIF	100.0	99.4
Overall	98.3	92.4

Table S13. Categorical and conditional agreements with APM and MGIT. Categorical and conditional agreements of CRyPTIC's microtitre plate MICs at day 14 with categorical and quantitative results obtained with MGIT and APM, along with sensitivity and specificity values for the drugs included in the panel for which suitable breakpoints are established, are shown below. Categorical agreement with MGIT results ranged from 100% for AMK, KAN, LEV, PAS, RFB, RIF to 89.5% for BDQ, DLM and EMB. Conditional agreement was higher than categorical for clofazimine. We also calculated the agreements with APM for all drugs, with the exception of PAS and ETH. The lowest categorical agreement was observed for CFZ, INH and MXF.

	APM								MGIT							
**drug**	**APM CC**	**CRYPTIC***	**AGAR***		**Sensitivity (95%CI)**	**Specificity (95%CI)**	**% Cat. Agr.**	**% Cond. Agr.**	**MGIT cutoff**	**CRYPTIC***	**MGIT**		**Sensitivity (95%CI)**	**Specificity (95%CI)**	**% Cat. Agr.**	**% Cond. Agr.**
			**R**	**S**							**R**	**S**				
AMK	2	R	2	0	100	100	100	100	1	R	3	0	100	100	100	100
		S	0	15	(34.2-100)	(79.6-100)				S	0	16	(43.9-100)	(80.6-100)		
BDQ	0.25	R	1	1	100	94.1	94.4	100	1	R	0	0	0	100	89.5	89.5
		S	0	16	(20.7-100)	(73-99)				S	2	17	(0-65.8)	(81.6-100)		
CFZ	0.5	R	0	1	0	94.1	88.9	94.4	1	R	0	0	0	100	89.5	94.7
		S	1	16	(0-79.3)	(73-99)				S	2	17	(0-65.8)	(81.6-100)		
DLM	0.06	R	2	0	100	100	100	100	0.12	R	1	1	50	94.1	89.5	89.5
		S	0	14	(34.2-100)	(78.5-100)				S	1	16	(9.5-90.5)	(73-99)		
EMB	5	R	3	0	75	100	94.4	94.4	5	R	2	1	66.7	93.8	89.5	89.5
		S	1	14	(30.1-95.4)	(78.5-100)				S	1	15	(20.8-93.9)	(71.7-98.9)		
ETH	5	R							5	R	3	0	75	100	94.7	94.7
		S								S	1	15	(30.1-95.4)	(79.6-100)		
INH	0.2	R	8	0	80	100	88.9	94.4	0.1	R	9	0	100	100	100	100
		S	2	8	(49-94.3)	(67.6-100)				S	0	10	(70.1-100)	(72.2-100)		
KAN	4	R	3	0	100	100	100	100	2.5	R	3	0	100	100	100	100
		S	0	15	(43.9-100)	(79.6-100)				S	0	16	(43.9-100)	(80.6-100)		
LEV	1	R	3	1	100	93.3	94.4	100	1	R	4	0	100	100	100	100
		S	0	14	(43.9-100)	(70.2-98.8)				S	0	15	(51-100)	(79.6-100)		
LZD	1	R	0	0		100	100	100	1	R	0	0	0	100	94.7	94.7
		S	0	18		(82.4-100)				S	1	18	(0-79.3)	(82.4-100)		
MXF	0.5	R	2	2	100	87.5	88.9	94.4	0.5	R	4	0	100	100	100	100
		S	0	14	(34.2-100)	(64-96.5)				S	0	15	(51-100)	(79.6-100)		
PAS	2	R							4	R	2	0	100	100	100	100
		S								S	0	16	(34.2-100)	(80.6-100)		
RFB	0.5	R	5	1	100	92.3	94.4	94.4	1	R	7	0	100	100	100	100
		S	0	12	(56.6-100)	(66.7-98.6)				S	0	12	(64.6-100)	(75.8-100)		
RIF	1	R	5	1	100	92.3	94.4	94.4	1	R	7	0	100	100	100	100
		S	0	12	(56.6-100)	(66.7-98.6)				S	0	12	(64.6-100)	(75.8-100)		

Table S14. Related to Table S12. The cells shaded grey are the drug/strain classification discrepancies between the reference method (APM or MGIT) and the mode MIC determined using the UKMYC plate. In each comparison the critical concentration (CC, for APM) or cutoff (for MGIT) is assumed to apply to the UKMYC5 plate. As one might expect many of MICs are close to the CC or cutoff and therefore the classification by UKMYC5 plate may change when critical concentrations are established.

			AGAR					MGIT			
drug	strain	Gene Mutations	APM CC	AGAR		UKMYC5		MGIT cut-off	MGIT R/S	UKMIC	
				mic	S/R	Mic	S/R			mic	S/R
BDQ	CRY-15; CRY-21	*Rv0678* Tyr92_STOP	0.25	0.12	S	0.5	R	1	R	0.5	S
CFZ	CRY-9; CRY-22	*Rv0678* Del A: Gln115_fs	0.5	0.5	S	1	R	1	R	1	S
CFZ	CRY-15; CRY-21	*Rv0678* Tyr92_STOP	0.5	1	R	0.25	S	1	R	0.25	S
EMB	CRY-14	*embB* Met306Ile	5	8	R	4	S	5	R	4	S
INH	CRY-4; CRY-30	Wildtype	0.2	0.4	R	0.05	S	0.1	S	0.05	S
INH	CRY-20	*prom fabG1-inhA* -15 c -> t	0.2	0.4	R	0.2	S	0.1	R	0.2	R
LEV	CRY-7; CRY-18	*gyrA* Ala90Val	1	0.25	S	2	R	1	R	2	R
MXF	CRY-2; CRY-25	*gyrA* Ala90Val	0.5	0.25	S	2	R	0.5	R	2	R
MXF	CRY-7; CRY-18	*gyrA* Ala90Val	0.5	0.25	S	1	R	0.5	R	1	R
RFB	CRY-7; CRY-18	*rpoB* Ser450Leu	0.5	0.5	S	4	R	1	R	4	R
RIF	CRY-1; CRY-17	*rpoB* Ser450Leu	1	1	S	8	R	1	R	8	R
BDQ	CRY-9; CRY-22	*Rv0678* Del A: Gln115_fs	0.25	0.5	R	0.5	R	1	R	0.5	S
DLM	CRY-9; CRY-22	*Rv1173* Arg536Leu	0.06	0.25	R	0.5	R	0.12	S	0.5	R
DLM	CRY-15; CRY-21	Wildtype	0.06	0.015	S	0.015	S	0.12	R	0.015	S
EMB	CRY-5; CRY10	*embB* Gln497Arg	5	8	R	16	R	5	S	16	R
ETH	CRY-1; CRY-17	Wildtype	5	NA	NA	2	S	5	R	2	S
LZD	CRY-5; CRY10	Wildtype	1	0.25	S	0.5	S	1	R	0.5	S

Table S15. Resistance patterns and genotype of the strains. The critical concentrations used for APM and MGIT are reported in Table S12. The high confidence mutations used to define strains as resistant to the relevant drug are highlighted in red – the other mutations are shown for information only. All non-synonymous mutations found in candidate resistance genes for DLM, BDQ, CLF, ETH, LZD, PAS are included in this list because the genetic basis for resistance to these drugs is not well characterized. Each APM or REMA result is the consensus of two measurements; in discrepant cases the lower value is chosen. This is a summary of Table S22.

Drug	Strain	MGIT	MIC APM	MIC REMA	gene	mutations
Amikacin	CRY-12; CRY-24	R	n/d	> 16	*rrs*	a1401g
	CRY-14	R	> 16 (R)	> 16	*rrs*	a1401g
	CRY-7; CRY-18	R	> 16 (R)	> 16	*rrs*	a1401g
Bedaquiline	CRY-12; CRY-24	S	0.015 (S)	0.5	*Rv0678*	Ins G: Arg140_fs
	CRY-15; CRY-21	R	0.12 (S)	2	*Rv0678*	Tyr92_STOP
	CRY-8; CRY-13	S	0.015 (S)	0.06	*Rv0678*	Val3Ile
	CRY-9; CRY-22	R	0.5 (R)	0.25	*Rv0678*	Del A: Gln115_fs
Clofazimine	CRY-12; CRY-24	S	0.03 (S)	0.5	*Rv0678*	Ins G: Arg140_fs
	CRY-15; CRY-21	R	1 (R)	1	*Rv0678*	Tyr92_STOP
	CRY-8; CRY-13	S	0.25 (S)	0.06	*Rv0678*	Val3Ile
	CRY-9; CRY-22	R	0.5 (S)	1	*Rv0678*	Del A: Gln115_fs
Delamanid	CRY-1; CRY-17	R	4 (R)	> 2	*ddn*	Trp88_ STOP
	CRY-9; CRY-22	S	0.25 (R)	2	*fbiC*	Arg536Leu
Ethambutol	CRY-14	R	8 (R)	4	*embB*	Met306Ile
	CRY-5; CRY-10	S	8 (R)	16	*embB*	Gln497Arg
	CRY-7; CRY-18	R	16 (R)	8	*embB*	Met306Val
	CRY-8; CRY-13	R	16 (R)	8	*embB*	Met306Ile
Ethionamide	CRY-20	R	n/d	n/d	*prom fabG1-inhA*	-15 c-> t
	CRY-5; CRY-10	R	n/d	n/d	*ethA*	Asp357Tyr
	CRY-5; CRY-10	R	n/d	n/d	*inhA*	Asn231Asp
	CRY-7; CRY-18	R	n/d	n/d	*ethA*	Del T: Lys37_fs
	CRY-7; CRY-18	R	n/d	n/d	*prom fabG1-inhA*	-34 c -> Del
Isoniazid	CRY-1; CRY-17	R	3.2 (R)	> 8	*katG*	Ser315Thr
	CRY-14	R	3.2 (R)	> 8	*katG*	Ser315Thr
	CRY-20	R	0.4 (R)	1	*prom fabG1-inhA*	-15 c -> t
	CRY-23	R	> 6.4 (R)	> 8	*katG*	Ser315Thr
	CRY-28	R	> 6.4 (R)	> 8	*katG*	Ser315Thr
	CRY-5; CRY-10	R	> 6.4 (R)	> 8	*katG*	Ser315Thr
	CRY-6; CRY-29	R	> 6.4 (R)	> 8	*katG*	Ser315Thr
	CRY-7; CRY-18	R	1.6 (R)	> 8	*katG*	Ser315Thr
	CRY-7; CRY-18	R	1.6 (R)	> 8	*prom fabG1-inhA*	-34 c -> Del
	CRY-8; CRY-13	R	> 6.4 (R)	> 8	*katG*	Ser315Thr
Kanamycin	CRY-12; CRY-24	R	> 16 (R)	> 32	*rrs*	a1401g
	CRY-14	R	16 (R)	> 32	*rrs*	a1401g
	CRY-7; CRY-18	R	8 (R)	> 32	*rrs*	a1401g

Levofloxacin	CRY-2; CRY-25	R	2 (R)	2	*gyrA*	Ala90Val
	CRY-5; CRY-10	R	8 (R)	8	*gyrA*	Ala90Val
	CRY-5; CRY-10	R	8 (R)	8	*gyrA*	Ser91Pro
	CRY-7; CRY-18	R	0.25 (S)	1	*gyrA*	Ala90Val
	CRY-8; CRY-13	R	4 (R)	2	*gyrA*	Ala90Val
Linezolid	CRY-16	S	0.5 (S)	0.25	*rrL*	c344t
	CRY-23	S	0.5 (S)	0.25	*rrL*	g1052t
	CRY-26	S	0.25 (S)	n/d	*rrL*	c344t
Moxifloxacin	CRY-2; CRY-25	R	0.25 (S)	0.25	*gyrA*	Ala90Val
	CRY-5; CRY-10	R	4 (R)	2	*gyrA*	Ala90Val
	CRY-5; CRY-10	R	4 (R)	2	*gyrA*	Ser91Pro
	CRY-7; CRY-18	R	0.25 (S)	0.25	*gyrA*	Ala90Val
	CRY-8; CRY-13	R	2 (R)	0.5	*gyrA*	Ala90Val
Para-aminosalicylic acid	CRY-23	R	n/d	n/d	*thyA*	Val261Gly
	CRY-9; CRY-22	NA	n/d	n/d	*folC*	Pro104Gln
Rifabutin	CRY-1; CRY-17	R	2 (R)	> 2	*rpoB*	Ser450Leu
	CRY-14	R	2 (R)	0.125	*rpoB*	Ser450Leu
	CRY-27	R	n/d	> 2	*rpoB*	Ser450Leu
	CRY-28	R	2 (R)	1	*rpoB*	Ser450Leu
	CRY-5; CRY-10	R	1 (R)	1	*rpoB*	Ser450Leu
	CRY-7; CRY-18	R	0.5 (S)	2	*rpoB*	Ser450Leu
	CRY-8; CRY-13	R	4 (R)	2	*rpoB*	Ser450Leu
Rifampicin	CRY-1; CRY-17	R	1 (R)	> 4	*rpoB*	Ser450Leu
	CRY-14	R	2 (R)	2	*rpoB*	Ser450Leu
	CRY-27	R	n/d	> 4	*rpoB*	Ser450Leu
	CRY-28	R	4 (R)	> 4	*rpoB*	Ser450Leu
	CRY-5; CRY-10	R	2 (R)	> 4	*rpoB*	Ser450Leu
	CRY-7; CRY-18	R	16 (R)	> 4	*rpoB*	Ser450Leu
	CRY-8; CRY-13	R	16 (R)	> 4	*rpoB*	Ser450Leu

Table S16. Mutations related to drug resistance identified by whole genome sequencing analysis of the EQA strains. *M. tuberculosis* H37Rv genome GenBank number NC_000962.3 was used as reference genome. The abbreviated lineages are: Euro American Super (EAS) lineage, East African-Indian (EAI) lineage and Latin American-Mediterranean (LAM) lineage. In red are indicated the high confidence mutations. Synonymous and phylogenetic SNPs were not considered for this analysis

		STRAIN (LINEAGE)																		
DRUGS	genes	CRY-1;17 (Beijing)	CRY-4;30 (EAS)	CRY-9;22 (EAI)	CRY 15;21 (EAI)	CRY-7;18 (Beijing)	CRY-5;10 (Beijing)	CRY-12;24 (Haarlem)	CRY-6;29 (Beijing)	CRY-14 (Beijing)	CRY-8;13 (LAM)	CRY-16	CRY-2;25 (EAI)	CRY-19 (Haarlem)	CRY-26 (EAS)	CRY-27 (Haarlem)	CRY-28 (LAM)	CRY-3;11	CRY-20 (LAM)	CRY-23 (EAI)
DLM	*ddn (Rv3547)*	**Trp88_STOP**	-	-	-	-	-	-	-	-	-	-	-	-	-	-	-	-	-	-
	fgd1 (Rv0407)	-	-	-	-	-	-	-	-	-	-	-	-	-	-	-	-	-	-	-
	fbiA (Rv3261)	-	-	-	-	-	-	-	-	-	-	-	-	-	-	-	-	-	-	-
	fbiB (Rv3262)	-	-	-	-	-	-	-	-	-	-	-	-	-	-	-	-	-	-	-
	fbiC (Rv1173)	-	-	Arg536Leu	-	-	-	-	-	-	-	-	-	-	-	-	-	-	-	-
BDQ	*atpE (Rv1305)*	-	-	-	-	-	-	-	-	-	-	-	-	-	-	-	-	-	-	-
BDQ-CLF	*Rv0678*	-	-	Del A: Gln115_fs	**Tyr92_STOP**	-	-	Ins G: Arg140_fs	-	-	Val3Ile	-	-	-	-	-	-	-	-	-
INH	*katG (Rv1908c)*	**Ser315Thr**	-	-	-	**Ser315Thr**	**Ser315Thr**	-	**Ser315Thr**	**Ser315Thr**	**Ser315Thr**	-	-	-	-	Phe332Val	**Ser315Thr**	-	-	**Ser315Thr**
INH-ETH	*fabG1 (Rv1483)*	-	-	-	-	-34 c -> Del	-	-	-60 c -> t	-	-	-	-	-	-	-	-	-	**-15 c -> t**	-
	inhA (Rv1484)	-	-	-	-	-	Asn231Asp	-	-	-	-	-	-	-	-	-	-	-	-	-
ETH	*ethA (Rv3854c)*	-	-	-	-	**Del T: Lys37_fs**	Asp357Tyr	-	-	-	-	-	-	-	-	-	-	-	-	-
RIF - RFB	*rpoB (Rv0667)*	**Ser450Leu**	-	-	-	**Ser450Leu**	**Ser450Leu**	-	-	**Ser450Leu**	**Ser450Leu**	-	-	-	-	**Ser450Leu**	**Ser450Leu**	-	-	-
EMB	*embB (Rv3795)*	Asp354Ala	-	-	-	**Met306Val**	**Gln497Arg**; Thr1082Ala	-	-	**Met306Ile**	**Met306Ile**	-	-	-	-	-	-	-	-	-
LEV-MXF	*gyrA (Rv0006)*	-	-	-	-	**Ala90Val**	**Ala90Val; Ser91Pro**	-	-	-	**Ala90Val**	-	**Ala90Val**	-	-	-	-	-	-	-
	gyrB (Rv0005)	-	-	-	Del CGC: pos 7159	-	-	-	-	-	-	-	-	-	-	-	-	-	-	-
AMK-KAN	*rrs (Rvnr01)*	-	-	-	-	**a1401g**	-	**a1401g**		**a1401g**	-	-	a514c	c1300t	-	-	-	-	-	a514c
	eis (Rv2416c)	-	-	-	-	-	-	-	-	-	-	-	-	-	-	-	-	-	-	-
LZD	*rplC (Rv0701)*	-	-	-	-	-	-	-	-	-	-	-	-	-	-	-	-	-	-	-
	rrL (Rvnr02)	-	-	-	-	-	-	-	-	-	-	c344t	-	-	c344t	-	-	-	-	g1052t
PAS	*thyA (Rv2764c)*	-	-		-	-	-	-	-	-	-	-	-	-	-	-	-	-	-	**Val261Gly**
	folC (Rv2447c)	-	-	Pro104Gln	-	-	-	-	-	-	-	-	-	-	-	-	-	-	-	-

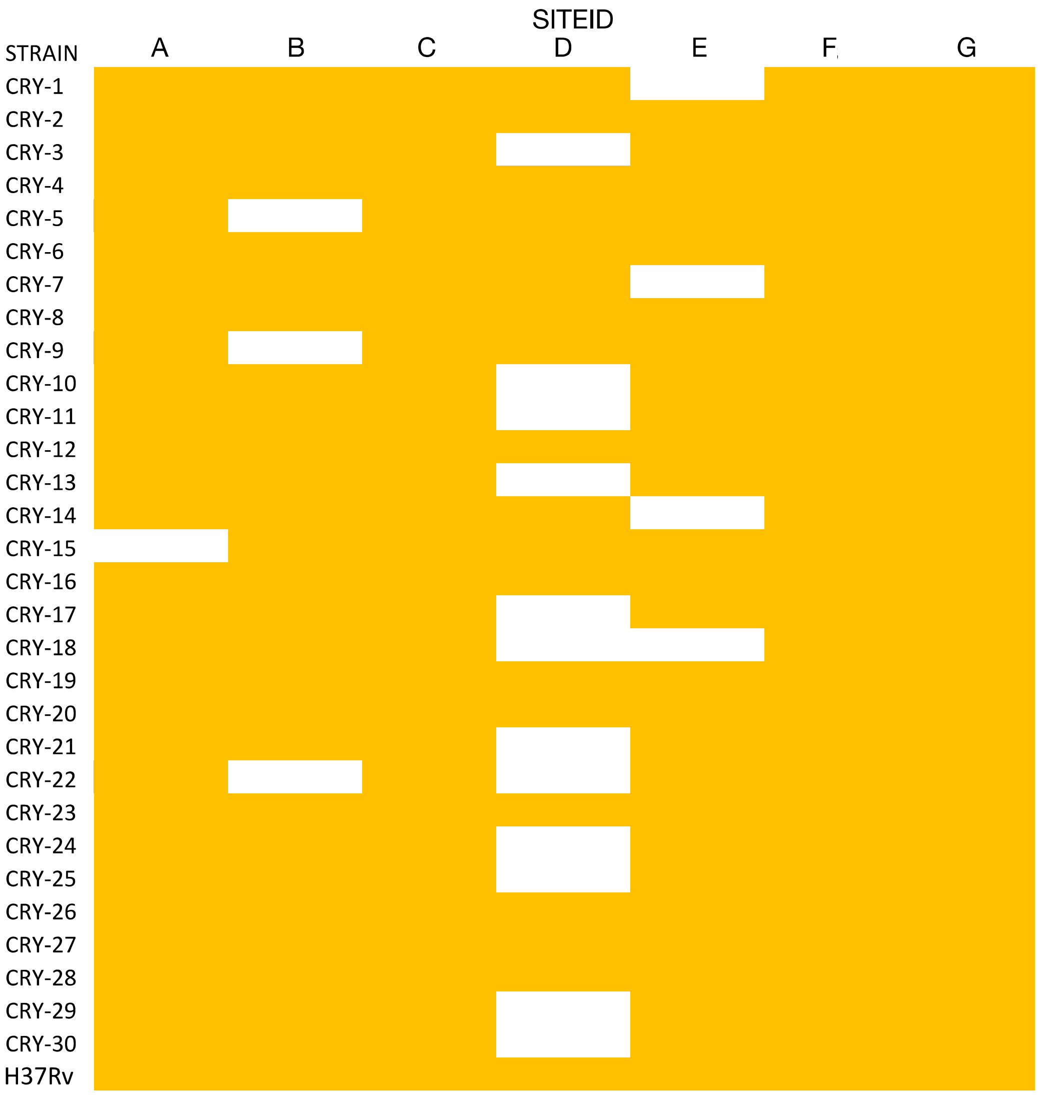

Figure S5. The seven participating laboratories received up to 31 vials of *M. tuberculosis* containing nineteen distinct external quality assurance (EQA) strains as described in the Material and Methods. A white rectangle indicates that site did not process that vial. Several sites, notable Site D, only received a subset of the vials for operational reasons.

Table S17. Additional data collected for all UKMYC5 plates tested. Taken from CRyPTIC Standard Operating Procedure for the Validation of a Dry-form Broth Microdilution Panel for TB DST (v1.1, 30 November 2016).

**Data Field**
Isolate ID, including replicate number
Reader name
Subculture date onto solid media
Inoculation date
Colony count on 7H10/7H11at day 21
Lot numbers of 7H9 with OADC, saline solution with tween and glass beads, microtitre plates
Reading dates
Information on the physical integrity of the plate
MIC readings using three different methods for each drug, i.e. mirrored box, Sensititre™ Vizion™ Digital Viewing System (Vizion) and inverted-light microscope
PZA DST inoculation date and result

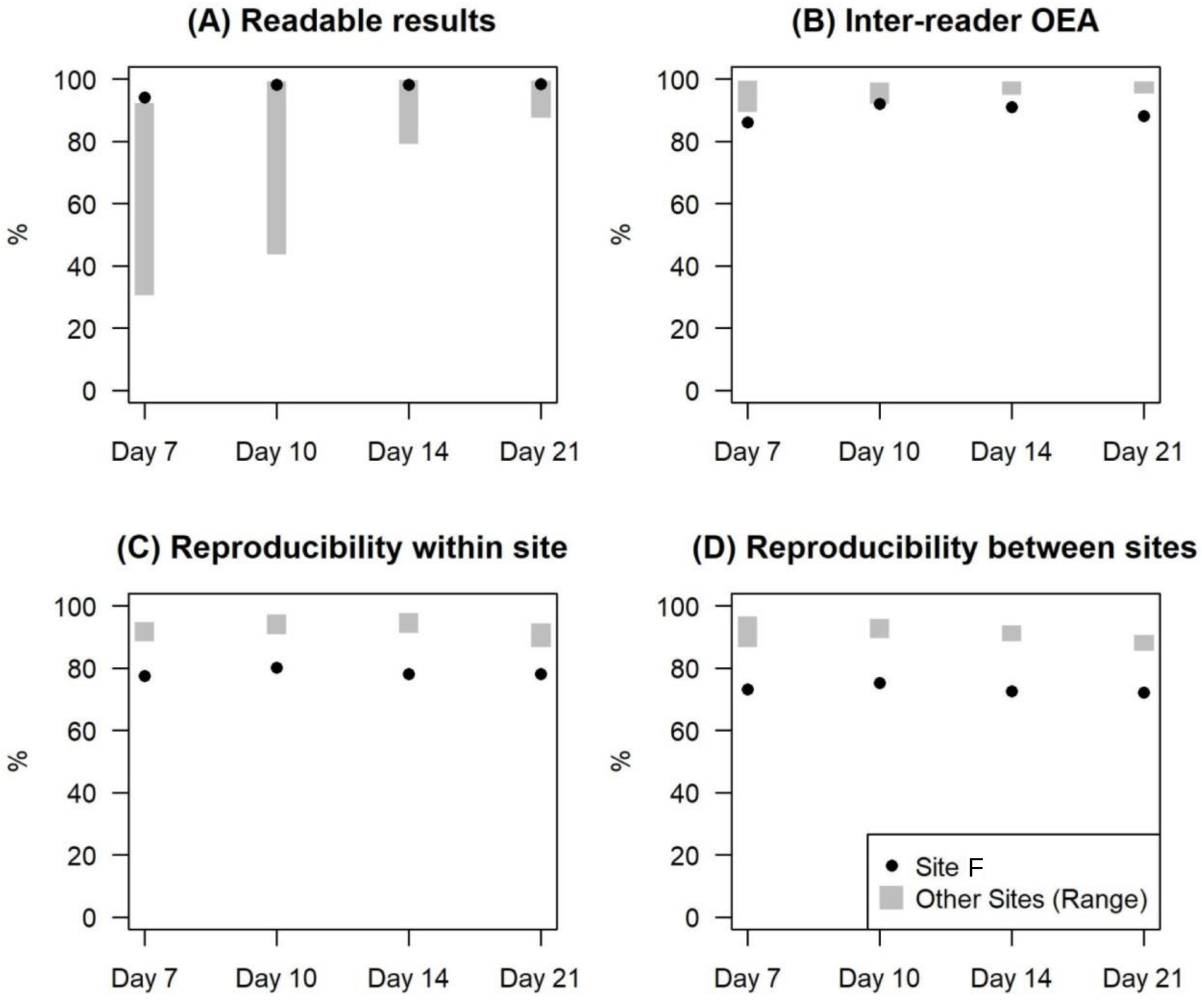

Figure S6. (**A**) The percentage of results that are considered readable increases with the incubation time. Site F showed a lower (**B**) inter-reader agreement and (**C**) intra- and (**D**) inter-laboratory reproducibility with respect to the other sites.

Table S18. Related to Figure S3**.** (**A**) The percentage of results that are considered readable increases with the incubation time. Site F showed (**B**) lower intra-reader agreement and lower (**C**) intra- and (**D**) inter-laboratory reproducibility with respect to the other sites.

(A) Percentage (%) of readable results

Reading Method	Day 7	Day 10	Day 14	Day 21
Site F	94.2	98.2	98.2	98.4
Other Sites (min-max)	(30.4-92.7)	(43.5-99.7)	(78.9-100.0)	(87.4-99.9)

(B) Inter-reader agreement (%)

Reading Method	Day 7	Day 10	Day 14	Day 21
Site F	86.2	92.0	91.0	88.3
Other Sites (min-max)	(89.2-99.8)	(91.8-99.3)	(94.8-99.8)	(95.2-99.7)

(C) Intra-laboratory reproducibility (%)

Reading Method	Day 7	Day 10	Day 14	Day 21
Site F	77.5	80.2	78.0	78.2
Other Sites (min-max)	(88.5-95.2)	(90.6-97.6)	(91.0-98.1)	(86.6-94.7)

(D) Inter-laboratory reproducibility (%)

Reading Method	Day 7	Day 10	Day 14	Day 21
Site F	73.2	75.3	72.5	72.2
Other Sites (min-max)	(86.6-97.1)	(89.5-96.1)	(88.3-94.2)	(85.2-91.1)

Table S19. Logistic mixed-effects models for inter-reader agreement, intra- and inter-laboratory reproducibility (adjusted for reading day, method, drug, interaction between reading day and method, interaction between reading day and drug). Random effects were defined nested with respect to strains and replicates.

	**Inter-reader agreement**			**Intra-laboratory reproducibility**			**Inter-laboratory reproducibility**		
**Parameter**	**Estimate**	**SE**	**p-value**	**Estimate**	**SE**	**p-value**	**Estimate**	**SE**	**p-value**
Intercept	3.634	0.246	<0.001	2.115	0.138	**<0.001**	1.909	0.133	**<0.001**
SITE ID									
SITE A vs F	0.422	0.166	0.011	1.252	0.134	**<0.001**	1.440	0.120	**<0.001**
SITE B vs F	3.265	0.266	**<0.001**	1.529	0.140	**<0.001**	1.560	0.124	**<0.001**
SITE C vs F	2.257	0.185	**<0.001**	2.043	0.135	**<0.001**	1.846	0.119	**<0.001**
SITE D vs F	2.765	0.273	**<0.001**	2.081	0.160	**<0.001**	1.326	0.137	**<0.001**
SITE E vs F	0.355	0.173	0.040	0.891	0.138	**<0.001**	1.173	0.123	**<0.001**
SITE G vs F	0.860	0.175	**<0.001**	1.287	0.134	**<0.001**	1.559	0.120	**<0.001**

Table S20. Serial drug concentrations used in the agar proportion method (APM)

Drug	Testing Range (mg/L)
Amikacin	0.06-16
Bedaquiline	0.004-4
Clofazimine	0.016-16
Delamanid	0.008-8
Ethambutol	0.06-32
Ethionamide	n/a
Isoniazid	0.008-8
Kanamycin	0.5-16
Levofloxacin	0.03-16
Linezolid	0.008-8
Moxifloxacin	0.016-16
Para-aminosalicylic acid	na
Rifabutin	0.004-4
Rifampicin	0.016-8

Table S21. Concentration ranges used in the resazurin microtitre assay (REMA).

Drug	Testing Range (mg/L)
Amikacin	0.03-16
Bedaquiline	0.004-2
Clofazimine	0.004-2
Delamanid	0.004-2
Ethambutol	0.03-16
Ethionamide	n/a
Isoniazid	0.016-8
Kanamycin	0.06-32
Levofloxacin	0.016-8
Linezolid	0.016-8
Moxifloxacin	0.008-4
Para-aminosalicylic acid	n/a
Rifabutin	0.004-2
Rifampicin	0.008-4

Table S22. See separate Excel spreadsheet.
